# Subfunctionalization of peroxisome proliferator response elements accounts for retention of duplicated *fabp1* genes in zebrafish

**DOI:** 10.1186/s12862-016-0717-x

**Published:** 2016-07-16

**Authors:** Robert B. Laprairie, Eileen M. Denovan-Wright, Jonathan M. Wright

**Affiliations:** Department of Pharmacology, Dalhousie University, 5850 College St, Halifax, NS B3H 4R2, Canada; Department of Biology, Dalhousie University, 31355 Oxford St, PO Box 15000, Halifax, NS B3H 4R2 Canada

**Keywords:** Peroxisome proliferator activated receptor (PPAR), Dual luciferase assay, Fatty acid-binding protein, Subfunctionalization, Neofunctionalization, Nonfunctionalization, Teleost fishes, Gene promoter evolution, Zebrafish, Spotted gar

## Abstract

**Background:**

In the duplication-degeneration-complementation (DDC) model, a duplicated gene has three possible fates: it may lose functionality through the accumulation of mutations (nonfunctionalization), acquire a new function (neofunctionalization), or each duplicate gene may retain a subset of functions of the ancestral gene (subfunctionalization). The role that promoter evolution plays in retention of duplicated genes in eukaryotic genomes is not well understood. Fatty acid-binding proteins (Fabp) belong to a multigene family that are highly conserved in sequence and function, but differ in their gene regulation, suggesting selective pressure is exerted *via* regulatory elements in the promoter.

**Results:**

In this study, we describe the PPAR regulation of zebrafish *fabp1a, fabp1b.1*, and *fabp1b.2* promoters and compare them to the PPAR regulation of the spotted gar *fabp1* promoter, representative of the ancestral *fabp1* gene. Evolution of the *fabp1* promoter was inferred by sequence analysis, and differential PPAR-agonist activation of *fabp1* promoter activity in zebrafish liver and intestine explant cells, and in HEK293A cells transiently transfected with wild-type and mutated *fabp1*promoter-reporter gene constructs. The promoter activity of spotted gar *fabp1,* representative of the ancestral *fabp1,* was induced by both PPARα- and PPARγ-specific agonists, but displayed a biphasic response to PPARα activation. Zebrafish *fabp1a* was PPARα-selective, *fabp1b.1* was PPARγ-selective, and *fabp1b.2* was not regulated by PPAR.

**Conclusions:**

The zebrafish *fabp1* promoters underwent two successive rounds of subfunctionalization with respect to PPAR regulation leading to retention of three zebrafish *fabp1* genes with stimuli-specific regulation. Using a pharmacological approach, we demonstrated here the divergent regulation of the zebrafish *fabp1a*, *fabp1b.1*, and *fabp1b.2* with regard to subfunctionalization of PPAR regulation following two rounds of gene duplication.

**Electronic supplementary material:**

The online version of this article (doi:10.1186/s12862-016-0717-x) contains supplementary material, which is available to authorized users.

## Background

Gene duplication is thought to facilitate increasing organismal complexity, but evolution does not accommodate redundancy. Duplication of genes can occur by unequal crossing-over during meiosis, replication slippage, retrotransposition, aneuploidy, or whole genome duplication [1]. The common fate of duplicated genes is loss of one copy owing to accumulated mutation and functional decay (non-functionalization) [2, 3]. Alternatively, both copies of a duplicated gene may be retained if one of the duplicates acquires a novel function (neo-functionalization), or the functions of the ancestral gene are subdivided between the duplicates (subfunctionalization) [2–4]. Non-, neo-, and sub-functionalization represent three possible fates of duplicated genes as described in the duplication degeneration complementation (DDC) model [2]. Both mutation of protein coding regions and the loss or gain of *cis-*acting regulatory elements in the promoters of duplicated genes may account for altered function of duplicated genes. Mutations in regulatory elements of promoters may affect tissue-, developmental stage- and stimulus-dependent transcript levels of duplicated genes [2–5].

Fatty acid binding proteins (Fabp), which belong to the multigene family of intracellular lipid-binding proteins, function as carriers of fatty acids, eicosanoids and other hydrophobic ligands to effectors in the cytosol and nucleus [6]. Previously, we observed that the promoters of the tandemly duplicated *fabp* genes of zebrafish, *fabp1b.1* and *fabp1b.2*, differ in their regulation by peroxisome proliferator activated receptors (PPARs), where *fabp1b.1* promoter activity was induced by PPAR, but *fabp1b.2* promoter activity was not induced by PPAR [7]. The zebrafish *fabp1a* and *fabp1b* genes were generated by duplication of ancestral *fabp1* gene owing to a whole genome duplication (WGD) event that occurred in the ray-finned teleost lineage approximately 325 mya [8–11]. Subsequently, the zebrafish *fabp1b.1* and *fabp1b.2* genes arose by tandem duplication of *fabp1b*, most likely by misaligned cross-over of homologous chromosomes during meiosis [12–14]. The zebrafish *fabp1b.1* and *fabp1b.2* genes are the only tandem duplicates of the multigene family of intracellular lipid-binding protein genes identified, thus far, in teleost fishes [12]. As a result, the zebrafish genome contains three extant *fabp1* genes, *fabp1a*, *fabp1b.1*, and *fabp1b.2*. Spotted gar (*Lepisosteus oculatus*, order *Lepisosteiformes*) belongs to an order of teleost fishes that did not undergo a WGD, therefore, its genome contains a single copy of the *fabp1* gene.

Zebrafish *fabp1b.1* and *fabp1b.2* differ in their responsiveness to dietary fatty acids: *fabp1b.1* mRNA levels are increased in the intestine of linolenic acid-fed zebrafish, whereas *fabp1b.2* mRNA levels are unaffected by linolenic acid [14]. Zebrafish *fabp1a, fabp1b.1*, and *fabp1b.2* also differ in their responsiveness to the non-selective PPAR agonist, clofibrate [15]. *fabp1a* mRNA levels are increased in the liver of clofibrate-fed zebrafish, *fabp1b.1* mRNA levels are increased in the heart of clofibrate-fed zebrafish, while *fabp1b.2* mRNA levels are unaffected by clofibrate [15]. These findings implicate the PPARs in the differential regulation of the *fabp1a*, *fabp1b.1* and *fabp1b.2* genes in zebrafish [14, 15].

PPARs are nuclear receptor transcription factors that bind, and are activated by, free fatty acids and eicosanoids [16–18]. Upon activation, PPARs heterodimerize with the retinoid X receptor (RXR) and bind to a PPAR response element (PPRE) located in the promoters of many vertebrate genes, including *fabp* genes [16–18]. The consensus sequence for the vertebrate PPRE is defined as 5′-CAAAACAGGTCANAGGTCA-3′ [16–18]. Binding of the PPAR to a PPRE may cause increased or decreased gene expression, depending on the gene [16–18]. Three PPAR isoforms have been identified across vertebrate species: PPARα, PPARγ, and PPARß/∂ [16–18]. While PPARα and PPARγ are expressed in many vertebrate tissues, PPARß/∂ expression is limited to the skin, adipose, and brain [16–18]. A PPRE may be PPAR isoform-selective (*i.e.,* a PPRE that preferentially binds PPARα relative to PPARγ) [16, 17]. A PPRE with high sequence identity in the 5′ flanking region (5′FR) (underlined: 5′-CAAAACAGGTCANAGGTCA-3′) to the consensus PPRE exhibits greater activation of transcription at promoters by the isoform PPARα compared to the isoform PPARγ, whereas PPARγ binding is less-dependent on the 5′FR than PPARα [16–18]. Both PPARα and PPARγ bind to the direct repeat element (DR1) (underlined 5′-CAAAACAGGTCANAGGTCA-3′) of the PPRE to activate transcription [16–18]. A PPRE with low sequence identity in the 5′FR and high sequence identity in the DR1, therefore, may be PPARγ-selective [16–18], as is apparent for *fabp1b.1* promoter activity, which displays PPARγ-selectivity in liver and intestine explant tissue and *fabp* promoter-reporter gene constructs in the human embryonic kidney cells, HEK293A [7].

The objective of this study was to investigate divergent, PPAR-dependent transcriptional regulation at the promoters of the zebrafish (*Danio rerio*) *fabp1a*, *fabp1b.1* and *fabp1b.2* genes, and the spotted gar *fabp1* gene (representative of the ancestral *fabp1* gene) in order to determine the molecular mechanisms that led to the retention of the three *fabp* genes in zebrafish following the teleost-specific WGD event and subsequent local (tandem) duplication event. To define teleost *fabp1* promoter evolution, the regulation of zebrafish *fabp1a, fabp1b.1,* and *fabp1b.2* gene promoters was investigated by three approaches: (1) assay of gene transcripts in liver and intestine explant cultures treated with PPAR-agonists; (2) identification of putative PPREs in the zebrafish *fabp1a, fabp1b.1,* and *fabp1b.* and the spotted gar *fabp1* promoters by *in silico* analysis; and (3) in HEK293A cells using wild-type and mutagenized zebrafish *fabp1a*, *fabp1b.1*, and *fabp1b.2*, and spotted gar *fabp1* promoters fused to the luciferase reporter gene, to determine the promoter-specific regulation of *fabp1* genes by PPARα and PPARγ. We applied a comparative pharmacological approach to spotted gar *fabp1* and zebrafish *fabp1a*, *fabp1b.1*, and *fabp1b.2* promoter activity across a wide range of PPAR agonist concentrations in the absence or presence of PPAR antagonists. In this way, it was possible to model evolutionary processes for PPAR isoform-selectivity through readily quantifiable measurements of agonist potency, efficacy, and specificity.

## Results

### Differential induction of zebrafish *fabp1a*, *fabp1b.1* and *fabp1b.2* transcription by PPAR agonists in zebrafish liver and intestine explant culture

**Fig. 1:**
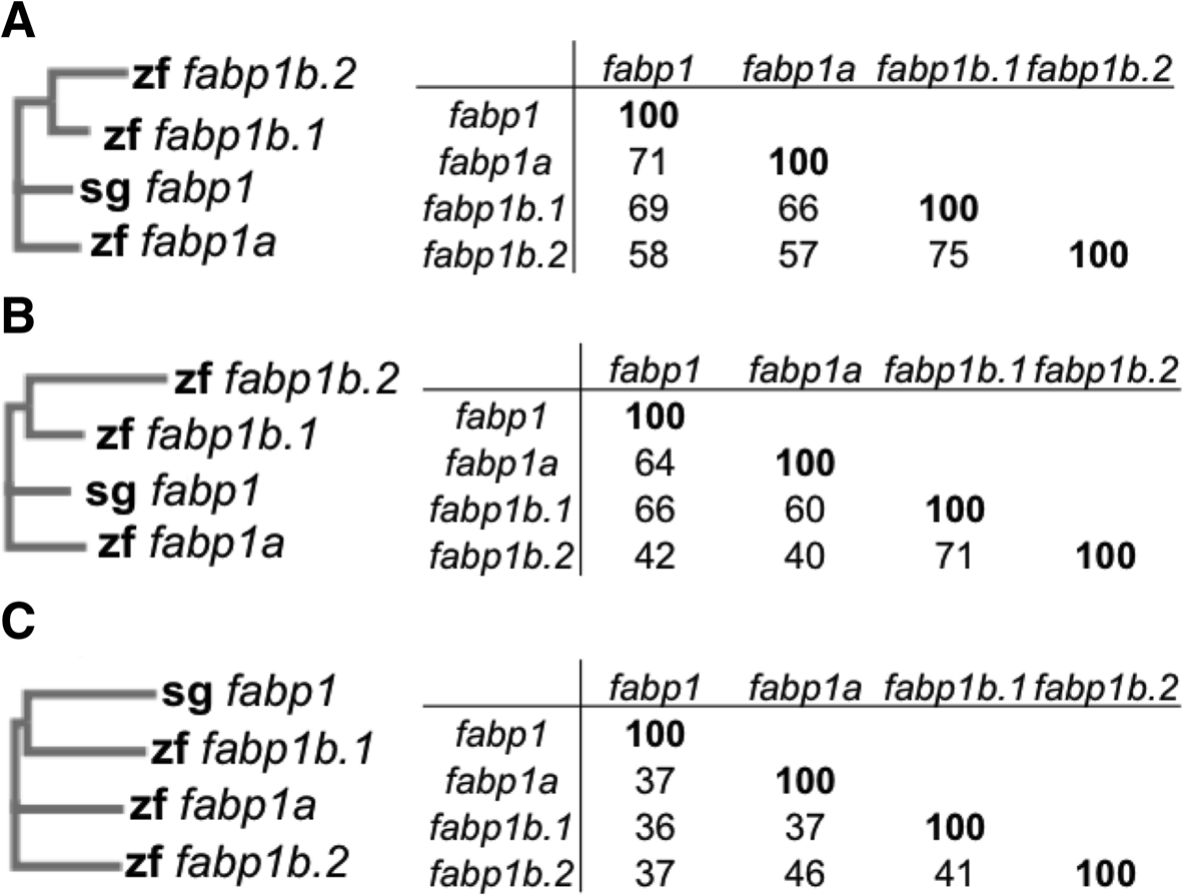
Cladograms and comparisons of sequence identity for spotted gar *fabp1* and zebrafish *fabp1a*, *fabp1b.1*, and *fabp1b.2* mRNA (**a**), amino acid (**b**), and promoter (**c**) sequences. Multiple sequence alignments were conducted using Clustal Omega with default settings. Cladograms of multiple sequence alignments are shown on the left (zf = zebrafish, sg = spotted gar). Tables shown on the right display percent sequence identity between genes. Accession files were: *fabp1* mRNA: XM_006626498, amino acid: XP_006626561, promoter: Gene ID: 102694982; *fabp1a* mRNA: NM_001044712, amino acid: NP_001038177, promoter: Gene ID: 791610; *fabp1b.1* mRNA: NM_001024651, amino acid: NP_001019822, promoter: Gene ID: 554095; *fabp1b.2* mRNA: XM_002663048, amino acid: XP_002663094, promoter: Gene ID: 100330224 from National Center for Biotechnology Information (NCBI) Gene [36]

The genomic organization of the *fabp1* gene of spotted gar and, the *fabp1a, fabp1b.1* and *fabp1b.2* genes of zebrafish is highly conserved; each gene consists of four exons, they share between 57 – 75 % mRNA sequence identity (Fig. 1a) and 40 – 71 % amino acid sequence identity (Fig. 1b), but differ markedly in their promoter sequences (Fig. 1c). The duplicated zebrafish *fabp1* genes also exhibit differential regulation by linolenic acid and by the PPAR-agonist, clofibrate [12, 14, 16].

**Fig. 2:**
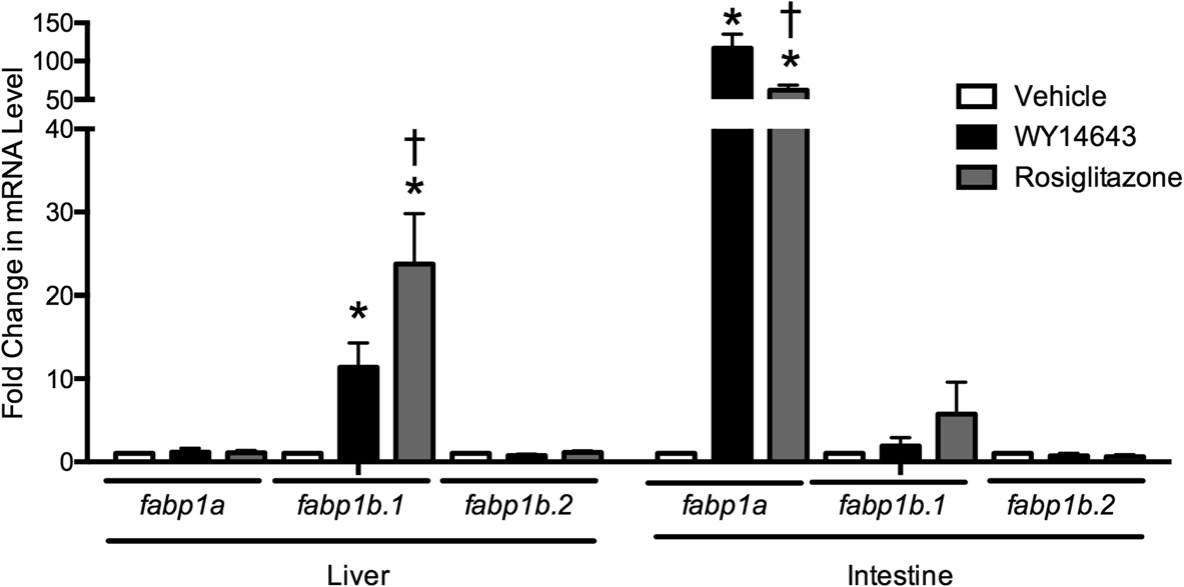
The PPAR-dependent induction of *fabp1a* and *fabp1b.1* mRNA was tissue-specific. Explant liver and intestine cells were cultured for 48 h before being treated with 1 μM WY14643 (PPARα-specific agonist) or rosiglitazone (PPARγ-specific agonist) for 24 h. *fabp1a*, *fabp1b.1*, and *fabp1b.2* mRNA levels were quantified by qRT-PCR using the ^ΔΔ^CT method and normalized to GAPDH. Data are mean ± SEM. *P < 0.001 compared to vehicle treatment within tissue and transcript, †*P* < 0.001 compared to WY14643 treatment within tissue and transcript as determined by two-way ANOVA followed by Bonferroni’s *post-hoc* test. *n* = 4

To determine if *fabp1a*, *fabp1b.1* and *fabp1b.2* mRNA levels are induced by PPAR activation, the steady-state levels of duplicated *fabp* transcript were quantified in explant tissue derived from zebrafish liver and intestine treated with the PPARα agonist, WY14,643, or the PPARγ agonist, rosiglitazone. *fabp1a* mRNA transcripts were detected in both liver and intestine. The PPARα agonist, WY14,643, increased *fabp1a* transcript levels 109-fold, while the PPARγ agonist, rosiglitazone, increased *fabp1a* transcript levels 60-fold. Induction of *fabp1a* transcriptional initiation by PPAR agonists was only in intestine explant tissue and not in liver explant tissue (Fig. 2). Transcriptional induction of the zebrafish *fabp1a*, therefore, appeared to be PPARα-selective as WY14643 resulted in higher *fabp1a* mRNA levels than rosiglitazone treatment (Fig. 2). *fabp1b.1* mRNA levels were increased 13-fold following WY14643 treatment, and 26-fold following rosiglitazone treatment in liver, but not intestine (Fig. 2). Based on the differential regulation of these duplicated genes by PPAR agonists, *fabp1b.1* induction appears PPARγ-selective as rosiglitazone treatment resulted in higher *fabp1b.1* mRNA levels than WY14643 treatment (Fig. 2). *fabp1b.2* mRNA was detected in both liver and intestine, but the steady-state level of *fabp1b.2* transcripts was not changed in liver or intestine explant tissue by either rosiglitazone or WY14643 treatment (Fig. 2). These data demonstrate that transcription of *fabp1a* and *fabp1b.1*, but not *fabp1b.2*, is induced by PPAR in zebrafish liver and intestine explant tissue.

### In silico analyses

Promoter sequences of the zebrafish *fabp1a*, *fabp1b.1,* and *fabp1b.2*, and spotted gar *fabp1* genes, 5′ of their transcription start sites (TSS), were analyzed *in silico* for the presence of putative PPREs. Sequences of 3,308 bp for zebrafish *fabp1a*, 3,059 bp for zebrafish *fabp1b.1*, 3,218 bp for zebrafish *fabp1b.2*, and 3,283 bp spotted gar *fabp1* were retrieved from databases using the conserved non-coding sequence (CNS) discovery pipeline (v. 3.0) [19] (Additional file 1) and putative PPREs identified by the algorithm, MatInspector (v. 8.1). The length (in bp) of the promoter fragments retrieved was chosen by the CNS discovery pipeline as the region within 4,000 bp 5′ upstream of the TSS containing > 60 % of transcription factor binding motifs with > 60 % sequence identity to the vertebrate transcription factor binding site [19].

**Fig. 3:**
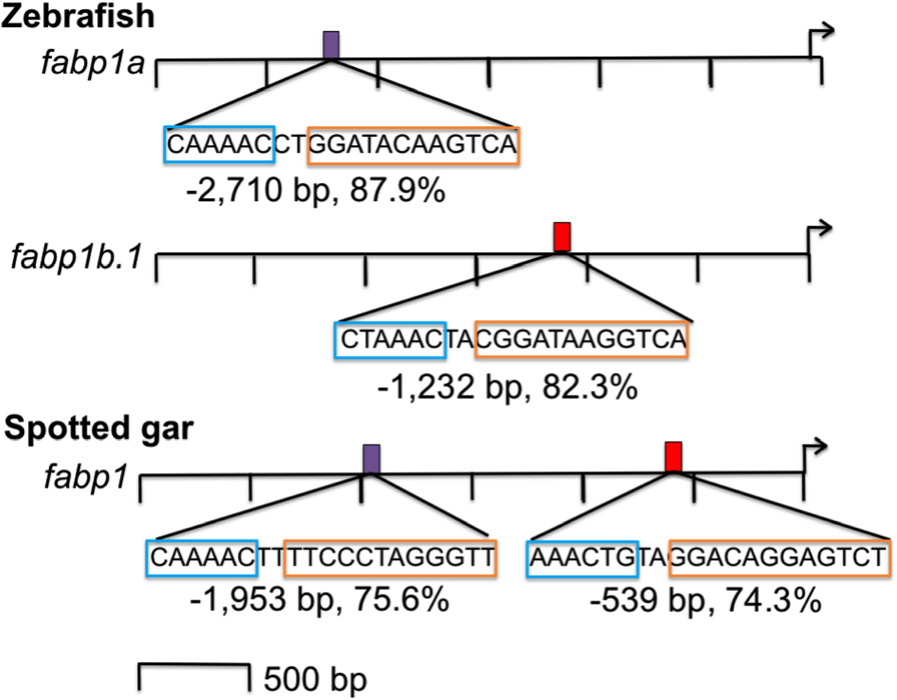
Putative PPREs in the zebrafish *fabp1a, fabp1b.1* and *fabp1b.2*, and spotted gar *fabp1* gene promoters. Putative PPREs were identified using MatInspector (v. 8.1). Approximately 3,000 bp promoter fragments are shown (scale: bars at 500 bp intervals). Right-facing arrows indicate the TSS. Gene name is indicated to the left of each promoter. Coloured rectangles indicate putative PPREs (sequence, position relative to TSS, and % sequence identity to the defined vertebrate PPRE consensus sequence below each promoter). Purple rectangles indicate PPREs that may be PPARα-selective. Red rectangles indicate PPREs that may be PPARγ-selective. Blue boxes indicate the 5′FR. Orange boxes indicate the DR1. A possible PPARα-selective PPRE was identified at −2,710 bp relative to the TSS of the zebrafish *fabp1a* promoter. A possible PPARγ-selective PPRE was identified at −1,232 bp relative to the TSS of the zebrafish *fabp1b.1* promoter. No PPREs were identified in the zebrafish *fabp1b.2* promoter. Two putative PPREs were identified at −1,953 bp (PPRE-1) and −539 bp (PPRE-2) relative to the TSS of the spotted gar *fabp1* promoter that were PPARα- and PPARγ-selective, respectively. Complete sequence data are provided in Additional file 1

Two putative PPREs were identified in the *fabp1a* promoter fragment that had 66.7 % and 87.9 % (indicated by the purple rectangle in Fig. 3) sequence identity to the consensus sequence for the vertebrate PPRE (5′ – 3′, respectively) (Additional file 1) [16, 17]. Five putative PPREs were identified in the *fabp1b.1* promoter fragment that exhibited 65 %, 68.1 %, 82.3 % (indicated by the red rectangle in Fig. 3), 65.1 % and 76.2 % sequence identity to the consensus sequence for the vertebrate PPRE (5′ – 3′, respectively) (Additional file 1) [16, 17]. No PPREs were identified within the *fabp1b.2* promoter fragment (Additional file 1)*.* The PPRE at −2,710 bp relative to the TSS in *fabp1a* displayed high sequence identity to the PPRE consensus in the 5′FR (5′-CAAAAC-3′), but not the DR1 (5′-AGGTCANAGGTCA-3′) region of the PPRE (Additional file 1). In contrast, the PPRE sequence at −1,232 bp relative to the TSS in *fabp1b.1* displayed high sequence identity to the vertebrate PPRE consensus in the DR1, but not 5′FR (Additional file 1). The 5′FR of the PPRE enhances the binding of PPARα to the PPRE and, thereby, imparts PPARα-selectivity to a PPRE. The 5′ FR is not required, however, for PPARγ binding to the PPRE and, thus, is not crucial for PPARγ-dependent gene regulation [16, 17]. Based on this observation, the putative PPRE identified at −2,710 bp in the *fabp1a* promoter appears to be PPARα-selective, whereas the PPRE at −1,232 bp in *fabp1b.1* was PPARγ-selective, which is consistent with the induction of the steady-state levels of *fabp1a* and *fabp1b.1* transcripts in zebrafish explant intestine cultures treated with PPARα- and PPARγ-specific agonists (Fig. 2).

Four putative PPREs were identified in the spotted gar *fabp1* promoter that had 75.6 % (indicated by the purple rectangle in Fig. 3), 64.7 %, 74.3 % (indicated by the red rectangle in Fig. 3), and 64.8 % sequence identity to the consensus sequence of the vertebrate PPRE (5′–3′, respectively). Similar to the PPRE in the zebrafish *fabp1a* gene, the putative PPRE present at −1,953 bp relative to the TSS of spotted gar *fabp1* displayed high sequence identity to the PPRE consensus sequence in the 5′FR (henceforth referred to as PPRE-1). Like the PPRE in zebrafish *fabp1b.1*, the putative PPRE at −539 bp relative to the TSS of spotted gar *fabp1* displayed high sequence identity to the PPRE consensus in the DR1 region (henceforth referred to as PPRE-2) [16, 17, 20, 21]. Taken together, these data suggest that the spotted gar *fabp1* promoter might be regulated by both PPARα- and PPARγ-selective PPREs [16, 17, 20, 21]. Furthermore, these data suggested that the PPREs with preferential binding affinity for PPARα and PPARγ, respectively, in the ancestral (spotted gar) *fabp1* gene were subdivided between *fabp1a* and *fabp1b.1* subsequent to the WGD event that occurred in ray-finned fish.

### Analyses of zebrafish *fabp1a*, *fabp1b.1* and *fabp1b.2* and spotted gar fabp1 promoter activity in HEK293A cells

**Fig. 4:**
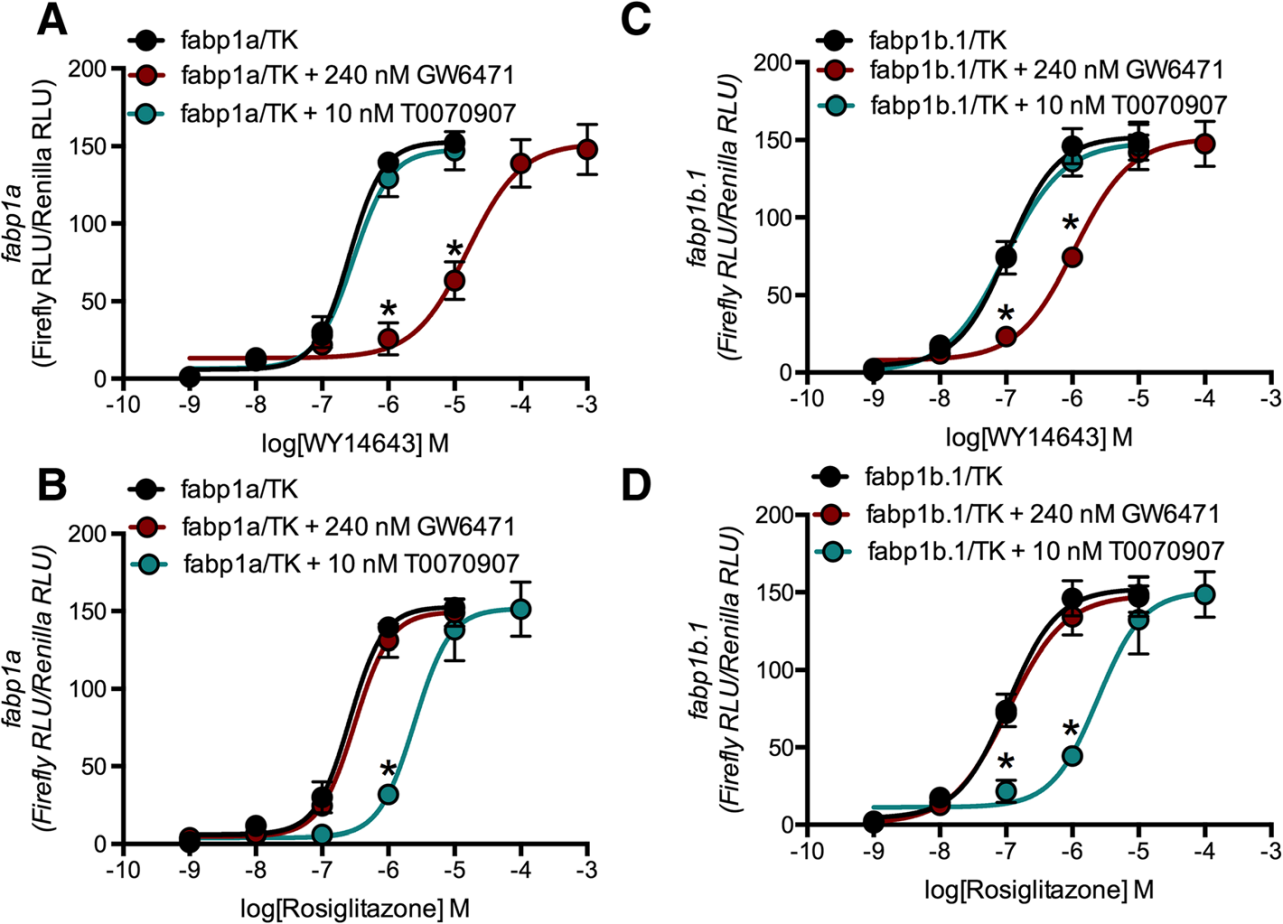
PPAR antagonism of zebrafish *fabp1a* and *fabp1b.1* promoter activity was PPARα- and PPARγ-selective, respectively. Firefly luciferase activity driven by the *fabp1a* (**a**, **b**) or *fabp1b.1* (**c**, **d**) promoters was normalized to *Renilla* luciferase activity driven by the TK promoter in HEK293A cells treated with 1 nM – 1 mM WY14643 (PPARα agonist) (**a**, **c**) or rosiglitazone (PPARγ agonist) (**b**, **d**) ± the PPAR antagonists 240 nM GW6471 (PPARα antagonist) or 10 nM T0070907 (PPARγ antagonist) for 24 h. Data are mean ± SD. *P < 0.001 compared to *fabp1a/*TK or *fabp1b.1*/TK alone as determined *via* one-way ANOVA followed by Bonferroni’s *post-hoc* test. *n* = 3

To determine functionality of putative PPREs in the *fabp1a* and *fabp1b.1* promoters, 3,300 bp of *fabp1a* and 2,847 bp of *fabp1b.1* 5′ upstream of their respective TSS were PCR-amplified from zebrafish genomic DNA and cloned into the pGL3-Basic promoter-reporter plasmid. In the pGL3-Basic plasmid, the 3,300 bp of *fabp1a* and 2,847 bp of *fabp1b.1* were fused to the firefly luciferase gene for functional promoter assays. All promoter fragments displayed similar basal promoter activity to the TK promoter in HEK293A cells (data not shown). Treatment of HEK293A cells transfected by the zebrafish *fabp1a* promoter construct with 1 nM – 1 mM WY14643 (PPARα agonist) for 24 h induced in *fabp1a* promoter activity (Fig. 4a). WY14643-dependent *fabp1a* promoter activity was inhibited by the PPARα-selective antagonist GW6471 [rightward shift in the concentration-response curve (CRC) and greater EC_50_] (Table 1, Fig. 4a). WY14643-dependent *fabp1a* promoter activity was not inhibited by the PPARγ-selective antagonist T0070907 (Table 1, Fig. 4a). Treatment of HEK293A cells transfected with the *fabp1a* promoter construct with 1 nM – 1 mM rosiglitazone (PPARγ agonist) for 24 h also increased *fabp1a* promoter activity (Fig. 4b). Rosiglitazone-dependent *fabp1a* promoter activity was inhibited by T0070907, but not by GW6471 (Table 1, Fig. 4b). WY14643 was a more potent PPAR agonist for induction of *fabp1a* promoter activity than rosiglitazone, indicating the zebrafish *fabp1a* gene contained at least one functional, PPARα-selective, PPRE (Table 1).

**Table 1:** Pharmacological characterization of PPAR induction of zebrafish *fabp1a* and *fabp1b.1* promoter activity

*fabp1a*										
	WY14643					Rosiglitazone				
	-	GW6471	T0070907	Δ5’FR	ΔDR1	-	GW6471	T0070907	Δ5’FR	ΔDR1
*E*_max_ (RLU)	152.80 ± 3.44	152.00 ± 7.80	147.90 ± 5.30	136.7 ± 11.1*	133.4 ± 4.94	152.80 ± 3.43	149.60 ± 3.66	151.70 ± 6.22	135.30 ± 7.11	144.40 ± 2.67
EC_50_ (μM)	0.26 (0.28–0.23)	15.7 (9.32–26.5)*	0.30 (0.20–0.44)	1.08 (0.71–6.70)*	11.9 (9.39–14.3)*	0.32 (0.30–0.34)***	0.31 (0.24–0.41)***	2.47 (1.59–3.83)**,***	10.6 (2.67–4.19)**	31.8 (17.1–43.6)**,***
*fabp1b.1*										
	WY14643					Rosiglitazone				
	-	GW6471	T0070907	Δ5’FR	ΔDR1	-	GW6471	T0070907	Δ5’FR	ΔDR1
*E*_max_ (RLU)	152.30 ± 5.57	151.30 ± 4.91	148.7 ± 5.27	146.4 ± 3.52	152.80 ± 7.24	152.30 ± 5.67	148.30 ± 4.87	150.70 ± 7.96	147.80 ± 2.02	178.50 ± 8.68**
EC_50_ (μM)	0.29 (0.17–0.49)	1.08 (0.86–1.54)*	0.10 (0.07–0.19)	0.67 (0.48–0.95)	28.8 (17.3–48.1)*	0.11 (0.07–0.15)***	0.11 (0.08–0.15)***	2.40 (1.39–4.15)**,***	0.70 (0.56–0.89)**	54.5 (32.9–90.4)**

**Fig. 5:**
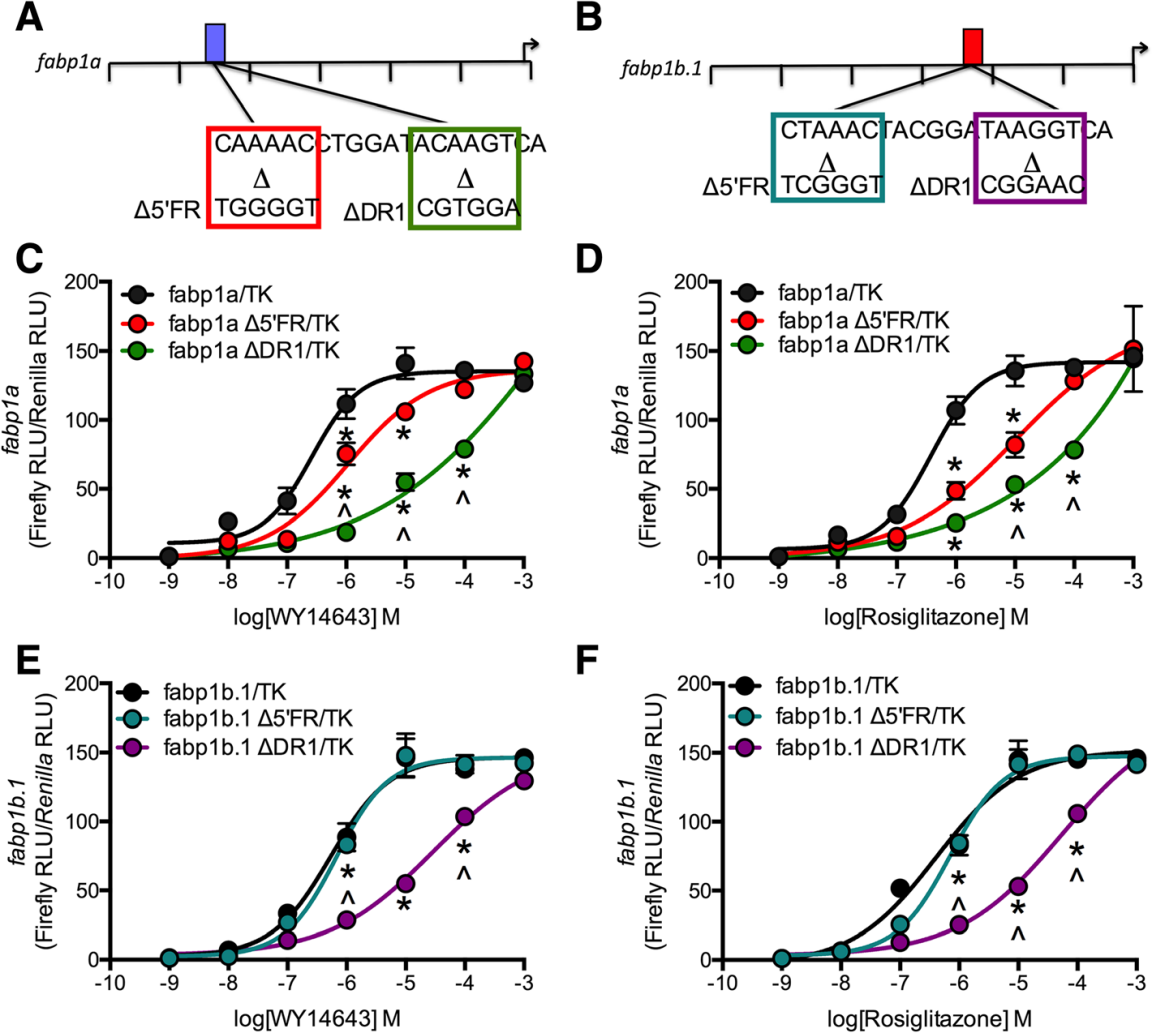
PPRE mutagenesis identified sites for PPARα- and PPARγ-selective induction of zebrafish *fabp1a* and *fabp1b.1* promoter activity, respectively. Site-directed mutagenesis was used to alter the 5′ flanking region (Δ5′FR) or direct repeat element (ΔDR1) PPAR binding sites in the *fabp1a* (**a**) and *fabp1b.1* (**b**) promoter fragments. Firefly luciferase activity driven by the *fabp1a* (**c**, **d**) or *fabp1b.1* (**e**, **f**) promoters was normalized to *Renilla* luciferase activity driven by the TK promoter in HEK293A cells treated with 1 nM – 1 mM WY14643 (PPARα agonist) (**c**, **e**) or rosiglitazone (PPARγ agonist) (**d**, **f)** for 24 h. Data are mean ± SD. *P < 0.001 compared to *fabp1a/*TK or *fabp1b.1*/TK alone, ^*P* < 0.001 compared to *fabp1a* Δ5′FR */*TK or *fabp1b.1* Δ5′FR /TK within agonist dose as determined *via* two-way ANOVA followed by Bonferroni’s *post-hoc* test. *n* = 3

Data derived from Figs. 4 and 5 presented as the mean ± SEM or 95 % confidence intervals (brackets)

*P < 0.05 compared to WY14643 treatment alone within promoter

**P < 0.05 compared to rosiglitazone treatment alone within promoter

***P < 0.05 rosiglitazone treatment compared to matched WY14643 treatment within promoter, as determined *via* two-way ANOVA followed by Bonferroni’s *post*-hoc analysis for *E*_max_, or by non-overlapping confidence intervals (EC_50_). *n* = 3

Treatment of HEK293A cells transfected by the *fabp1b.1* promoter construct with WY14643 induced *fabp1b.1* promoter activity (Fig. 4c). WY14643-dependent *fabp1b.1* promoter activity was inhibited by GW6471, but not by T0070907 (Table 1, Fig. 4c). Treatment of HEK293A cells transfected by the *fabp1b.1* promoter construct with rosiglitazone also increased *fabp1b.1* promoter activity (Fig. 4d). Rosiglitazone-induced *fabp1b.1* promoter activity was inhibited by T0070907, but not by GW6471 (Table 1, Fig. 4d). Rosiglitazone was a more potent agonist of *fabp1b.1* promoter activity than WY14643, indicating that the zebrafish *fabp1b.1* promoter contained a functional, PPARγ-selective PPRE (Table 1). Efficacy (*E*_max_) did not differ between agonists or promoters. Thus, differences in agonist activity between the *fabp1a* and *fabp1b.1* promoters were attributed to disparate potencies (*i.e.* EC_50_) of the PPAR isoforms acting at the *fabp1a* and *fabp1b.1* promoter PPREs (Table 1).

Site-directed mutagenesis was used to confirm the functionality of putative PPREs identified in the zebrafish *fabp1a* and *fabp1b.1* promoters. Mutagenesis of the 5′FR was expected to reduce the affinity of PPARα to the PPRE (*i.e.* a rightward shift in the CRC), whereas mutagenesis of the DR1 was expected to generally reduce PPAR affinity to the promoter [16–18]. The 5′FR and DR1 elements of the *fabp1a* PPRE (−2,710 bp) and the *fabp1b.1* PPRE (−1,232) were individually mutagenized (Fig. 5a, b).

Treatment of HEK293A cells transfected by the *fabp1a* Δ5′FR promoter construct with WY14643 shifted the CRC to the right compared to non-mutated *fabp1a* (Table 1, Fig. 5c). Treatment of HEK293A cells transfected with the *fabp1a* ΔDR1 promoter construct with WY14643 shifted the CRC to the right compared to non-mutated *fabp1a* and *fabp1a* Δ5′FR (Table 1, Fig. 5c). Treatment of HEK293A cells transfected by the *fabp1a* Δ5′FR promoter construct with rosiglitazone shifted the CRC compared to non-mutated *fabp1a* (Table 1, Fig. 5d). The CRC was shifted to the right in HEK293A cells transfected with the *fabp1a* ΔDR1 promoter construct and treated with rosiglitazone compared to non-mutated *fabp1a* and and *fabp1a* Δ5′FR (Table 1, Fig. 5d). These data demonstrate that the PPRE at −2,710 was functional and regulated, in part, by PPARα as mutagenesis of the 5′FR consistently affected PPAR-dependent induction of promoter activity [17]. Moreover, the DR1 element of the PPRE is required for the binding of all PPARs, a finding further supported by the functionality of the PPRE at −2,710 bp of the *fabp1a* promoter fragment [16–18].

WY14643 did not change the CRC for promoter activity in HEK293A cells transfected by the *fabp1b.1* Δ5′FR promoter construct compared to non-mutated *fabp1b.1* promoter construct (Table 1, Fig. 5e). WY14643 treatment shifted the CRC to the right in HEK293A cells transfected by the *fabp1b.1* ΔDR1 promoter construct compared to non-mutated *fabp1b.1* promoter (Table 1, Fig. 5e). Although the EC_50_ was shifted slightly to the right, the rosiglitazone produced a potent and fully efficacious response in HEK293A cells transfected by the *fabp1b.1* Δ5′FR promoter compared to non-mutated *fabp1b.1* promoter (Table 1, Fig. 5f). The rosiglitazone CRC was shifted to the right in HEK293A cells transfected with the *fabp1b.1* ΔDR1 promoter construct by 2.5 orders of magnitude compared to non-mutated *fabp1b.1* promoter (Table 1, Fig. 5e). These observations provide compelling evidence that the zebrafish *fabp1b.1* promoter region contains a functional, PPARγ-selective PPRE at −1,232 bp as rosiglitazone was a more potent agonist of PPAR induction of *fabp1b.1* promoter activity than WY14643, and the DR1 element, not the 5′FR, was the major regulator of PPAR potency in these assays (Table 1). Since neither mutagenesis of the 5′FR or DR1 in *fabp1a* or *fabp1b.1* abolished transcriptional induction of these *fabp* genes by PPAR agonism, and no change in *E*_max_ was observed, additional, functional PPREs are likely present in both the *fabp1a* and *fabp1b.1* promoters.

**Fig. 6:**
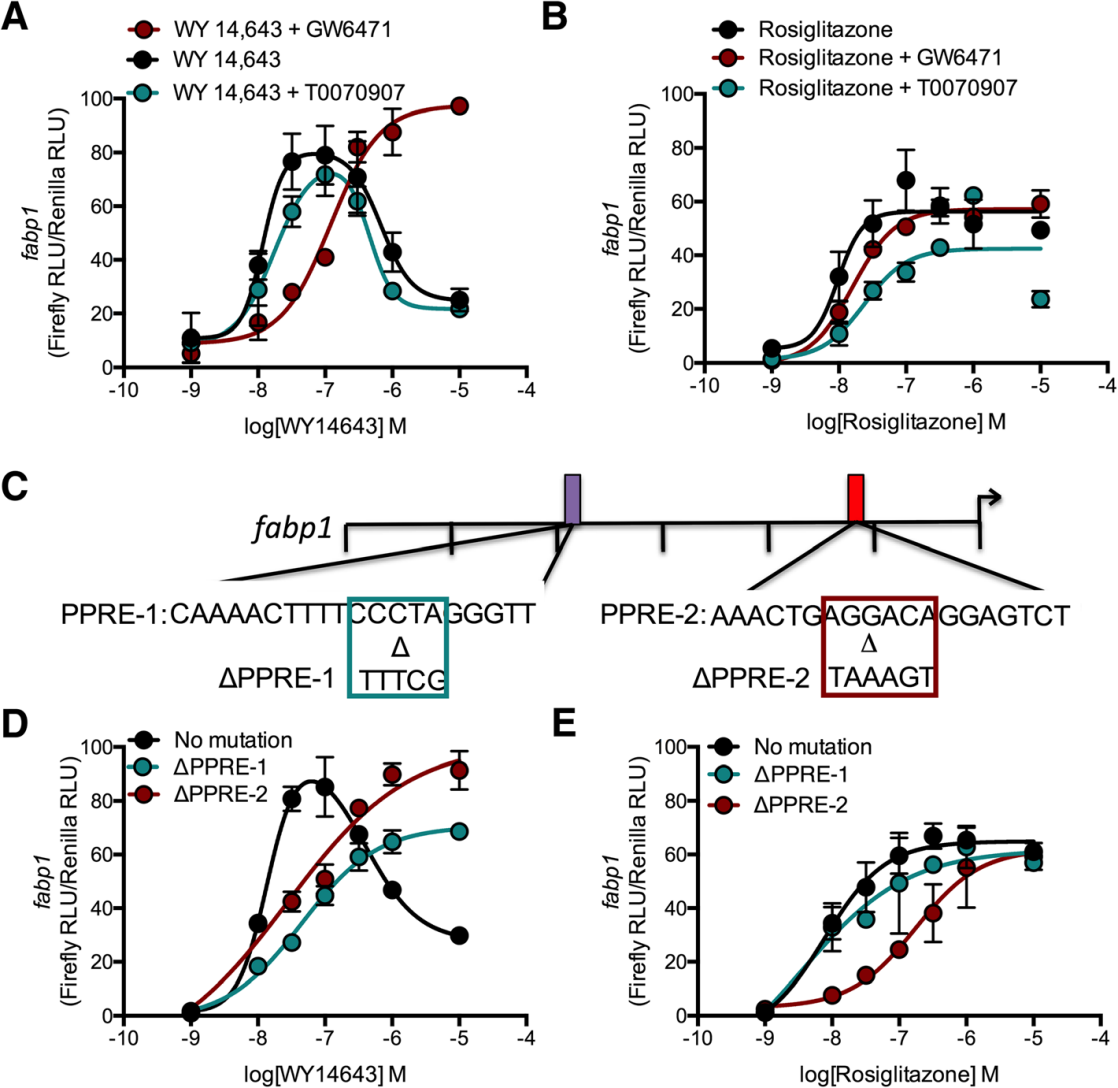
PPAR induction of the spotted gar *fabp1* promoter was PPARα- and PPARγ-selective. **a**,**b**) Firefly luciferase activity driven by the *fapb1* promoter normalized to *Renilla* luciferase activity driven by the TK promoter in HEK293A cells treated with 1 nM – 10 μM WY14643 (PPARα agonist) (**a**) or rosiglitazone (PPARγ agonist) (**b**) ± 240 nM GW6471 (PPARα antagonist) or 10 nM T0070907 (PPARγ antagonist) for 24 h. **C**) Site-directed mutagenesis was used to alter the DR1 PPAR binding sites of two *fabp1* PPREs at −1,953 bp (ΔPPRE-1) or −539 bp (ΔPPRE-2). **d**, **e**) Firefly luciferase activity driven by the *fapb1* ‘No mutation’, ΔPPRE-1, or ΔPPRE-2 promoters normalized to *Renilla* luciferase activity driven by the TK promoter in HEK293A cells treated with 1 nM – 10 μM WY14643 (**d**) or rosiglitazone (**e**). Data are mean ± SD. *P < 0.001 compared to agonist alone or ‘No mutation’, as determined *via* two-way ANOVA followed by Bonferroni’s *post-hoc* test. *n* = 3 – 4

Given that both *fabp1a* and *fabp1b.1* promoters contained functional, PPAR subtype-selective PPREs, we assayed the responsiveness of the orthologous spotted gar *fabp1* gene promoter activity to PPAR isoform-specific agonisism. A 3,283 bp fragment of the spotted gar *fabp1* promoter, 5′ upstream of its TSS, was PCR-amplified from spotted gar genomic DNA and cloned into the pGL3-Basic promoter-reporter plasmid 5′ of the firefly luciferase gene. Treatment of HEK293A cells transfected by the *fabp1* promoter construct with WY14643 resulted in a bell-shaped CRC for *fabp1* promoter activity (Table 2, Fig. 6a). Co-treatment with WY14643 and T0070907 did not change CRC of spotted gar *fabp1* promoter activity (Table 2, Fig. 6a). Co-treatment with WY14643 and GW6471 abolished the bell-shape of the *fabp1* CRC and shifted the slope of the CRC to the right compared to the upward slope of the WY14643 CRC (Table 2, Fig. 6a). In contrast to WY14643, treatment of HEK293A cells (transfected with the *fabp1* promoter) with rosiglitazone resulted in a concentration-dependent increase in *fabp1* promoter activity (Table 2, Fig. 6b). The CRC for *fabp1* promoter activity in cells treated with rosiglitazone was not changed by co-treatment with GW6471, but was shifted to the right by co-treatment with T0070907 (Table 2, Fig. 6b). Based on the results of these functional assays, we conclude that spotted gar *fabp1* promoter activity was inducible by both PPARα and PPARγ. However, higher concentrations of WY14643 (PPARα agonist), but not rosiglitazone (PPARγ agonist), reduced promoter activity of the spotted gar *fabp1* genes suggesting an inhibitory effect of PPARα. This inhibitory effect of the PPARα agonist may be mediated by recruitment of a transcriptional repressor to the PPRE-bound PPARα. Alternatively, this inhibitory effect may be mediated by steric hindrance of PPARα (*i.e.* a non-specific cross-activation of PPARγ at high concentrations of WY1463).

**Table 2:** Pharmacological characterization of PPAR agonist and antagonist regulation of spotted gar *fabp1* promoter activity

	WY14643					
	Upward Slope			Downward Slope		
	-	GW6471	T0070907	-	GW6471	T0070907
*E*_max_ (RLU)	80.6 ± 7.40	97.5 ± 3.78	74.2 ± 9.63	-	-	-
*E*_min_ (RLU)	10.9 ± 4.64	8.86 ± 3.50	8.93 ± 4.72	24.7 ± 4.88**	-	21.6 ± 4.13**
EC_50_ (μM)	0.12 (0.07–0.28)	0.54 (0.43–0.65)*	0.17 (0.05–0.25)	0.69 (0.52–1.1)**	–	0.45 (0.22–0.71)**
	Rosiglitazone					
	Upward Slope			Downward Slope		
	-	GW6471	T0070907	-	GW6471	T0070907
*E*_max_ (RLU)	56.3 ± 2.40	57.4 ± 2.44	42.5 ± 4.50	-	-	-
*E*_min_ (RLU)	5.28 ± 4.80	−0.42 ± 2.73	1.21 ± 7.80	-	-	-
EC_50_ (μM)	0.009 (0.006–0.017)	0.016 (0.012–0.021)	0.024 (0.021–0.062)*	-	-	-

Data derived from Fig. 6 presented as the mean ± SEM or 95 % confidence intervals (brackets)

*P < 0.05 compared to agonist treatment alone within the upward slope

**P < 0.05 compared to matched treatment between upward and downward slopes, as determined *via* two-way ANOVA followed by Bonferroni’s *post*-hoc analysis for *E*_max_, *E*_min_, or by non-overlapping confidence intervals (EC_50_). *n =* 3*–*4

The two putative PPREs were identified in the spotted gar *fabp1* promoter region, one located at −1,953 bp 5′ upstream of the TSS (PPRE-1), a location similar to the functional PPRE identified in the zebrafish *fabp1a*, and a second PPRE located at −539 bp (PPRE-2), in approximately the same location of a functional PPRE identified in the zebrafish *fabp1b.1* promoter. Site-directed mutagenesis was used to confirm the functionality of these putative PPREs in the spotted gar *fabp1* gene. The DR1 regions of the PPRE-1 and PPRE-2 were individually mutagenized (ΔPPRE-1, ΔPPRE-2); both mutagenized PPREs reduced PPAR-induced promoter activity of the spotted gar *fabp1* promoter (Fig. 6c) [16, 17]. ΔPPRE-1 or ΔPPRE-2 of the spotted gar *fabp1* abolished the bell-shaped CRC of promoter activity observed following WY14643 treatment of the transfected HEK293A cells (Table 3, Fig. 6d). ΔPPRE-1 or ΔPPRE-2 of *fabp1* also shifted either curve to the right compared to the upward slope of the non-mutated *fabp1* promoter (Table 3, Fig. 6d). WY14643 induced spotted gar ΔPPRE-1 *fabp1* promoter activity much more than the promoter activity of the spotted gar ΔPPRE-2 *fabp1* promoter activity (Table 3, Fig. 6d). The ΔPPRE-2 *fabp1* promoter rosiglitazone CRC was shifted to the right compared to the non-mutated *fabp1* promoter (Table 3, Fig. 6e). The ΔPPRE-1 *fabp1* promoter and non-mutated *fabp1* promoter displayed similar responses to rosiglitazone (Table 3, Fig. 6e). We conclude from these functional promoter assays using the PPARα- and the PPARγ-specific agonists, that PPRE-1 on the spotted gar *fabp1* promoter was PPARα-selective and mediated both increases (concentrations of PPAR agonist < 100 nM) and decreases (concentrations of PPAR agonist > 100 nM) in promoter activity. PPRE-2 was PPARγ-selective.

**Table 3:** Pharmacological characterization of spotted gar *fabp1* promoter PPRE mutants

	WY14643					
	Upward Slope			Downward Slope		
	No mutation	ΔPPRE-1	ΔPPRE-2	No mutation	ΔPPRE-1	ΔPPRE-2
*E*_max_ (RLU)	101 ± 2.56	70.6 ± 2.17*	102 ± 8.65	-	-	-
*E*_min_ (RLU)	1.32 ± 2.91	–1.64 ± 3.08	–17.4 ± 18.2	27.6 ± 4.76**	-	-
EC_50_ (μM)	0.013 (0.004–0.023)	0.045 (0.032–0.064)*	0.028 (0.008–0.098)	0.38 (0.20–0.41)**	-	-
	Rosiglitazone					
	Upward Slope			Downward Slope		
	No mutation	ΔPPRE-1	ΔPPRE-2	No mutation	ΔPPRE-1	ΔPPRE-2
*E*_max_ (RLU)	64.9 ± 2.34	61.9 ± 6.66	62.1 ± 5.07	-	-	-
*E*_min_ (RLU)	−7.47 ± 3.79	1.17 ± 0.05	2.94 ± 4.03	-	-	-
EC_50_ (μM)	0.008 (0.003–0.019)	0.003 (0.001–1.34)	0.18 (0.08–0.36)*	-	-	-

Data derived from Fig. 6 presented as the mean ± SEM or 95 % confidence intervals (brackets)

*P < 0.05 compared to agonist treatment alone within the upward slope

**P < 0.05 compared to matched treatment between upward and downward slopes, as determined *via* two-way ANOVA followed by Bonferroni’s *post*-hoc analysis for *E*_max_, *E*_min_, or by non-overlapping confidence intervals (EC_50_). *n* = 3

## Discussion

In this study, we employed a pharmacological approach to define the PPAR selectivity, potency, and efficacy of PPAR-dependent regulation in the promoters of the zebrafish *fabp1a, fabp1b.1* and *fabp1b.2* genes. We observed that the zebrafish *fabp1a* promoter contained a functional, PPARα-selective PPRE, while the zebrafish *fabp1b.1* promoter contained a functional, PPARγ-selective PPRE. The spotted gar *fabp1* promoter contained two functional PPREs: a PPARα-selective PPRE (PPRE-1) and a PPARγ-selective PPRE (PPRE-2). These results are consistent with previously published conclusions that: (1) the steady-state level of *fabp1a* and *fabp1b.1* mRNA and hnRNA levels are induced in adult zebrafish fed a linolenic acid- or clofibrate-rich diets, and this transcriptional activation is mediated by PPAR [14, 15], and (2) that the *fabp1a* and *fabp1b.1* promoters are functionally-selective for PPARα and PPARγ, respectively as described here and in a previous report [7].

The spotted gar *fabp1* promoter served as a representative of the ancestral *fabp1* gene that gave rise to *fabp1a* and *fabp1b* following the teleost WGD [9]. The two functional PPREs identified in the spotted gar *fabp1* promoter were oriented such that PPRE-1 was PPARα-selective and PPRE-2 was PPARγ-selective. Pharmacological analyses and site-directed mutagenesis demonstrated that both the spotted gar PPRE-1 of *fabp1* and the similarly-aligned zebrafish PPRE of *fabp1a* (Fig. 1) were more responsive to PPARα-agonists and antagonists than to PPARγ-agonists and antagonists, based on promoter activity assays, suggesting that the *fabp1a* PPRE at −1,953 bp was derived from the PPARα-selective PPRE-1 in the ancestral *fabp1* prior to the teleost WGD. Furthermore, pharmacological analyses and site-directed mutagenesis demonstrated that both the PPRE-2 of spotted gar *fabp1* and the similarly-aligned zebrafish PPRE of *fabp1b.1* (Fig. 1) were both more responsive to PPARγ agonists and antagonists than to PPARα agonists and antagonist as assayed by the induction of promoter activity, suggesting that the *fabp1b.1* PPRE at −539 bp was derived from an ancestral PPARγ-selective PPRE-2 present in the spotted gar (ancestral) *fabp1* gene prior to its duplication following the teleost WGD.

Previous studies have focused on non-quantitative or semi-quantitative data derived from electrophoretic mobility shift assays to determine the specificity of PPARs interaction with PPREs [16–18, 22]. The unique pharmacological approach used in this study to define the regulation and its evolution of promoter activity provided quantitative data, which supports the contention that the 5′FR is directly involved in PPARα-, but not PPARγ-, dependent promoter activation [17]. Furthermore, this work supports earlier findings that the DR1 regulates general PPAR-dependent promoter activation [16–18, 22].

**Fig. 7:**
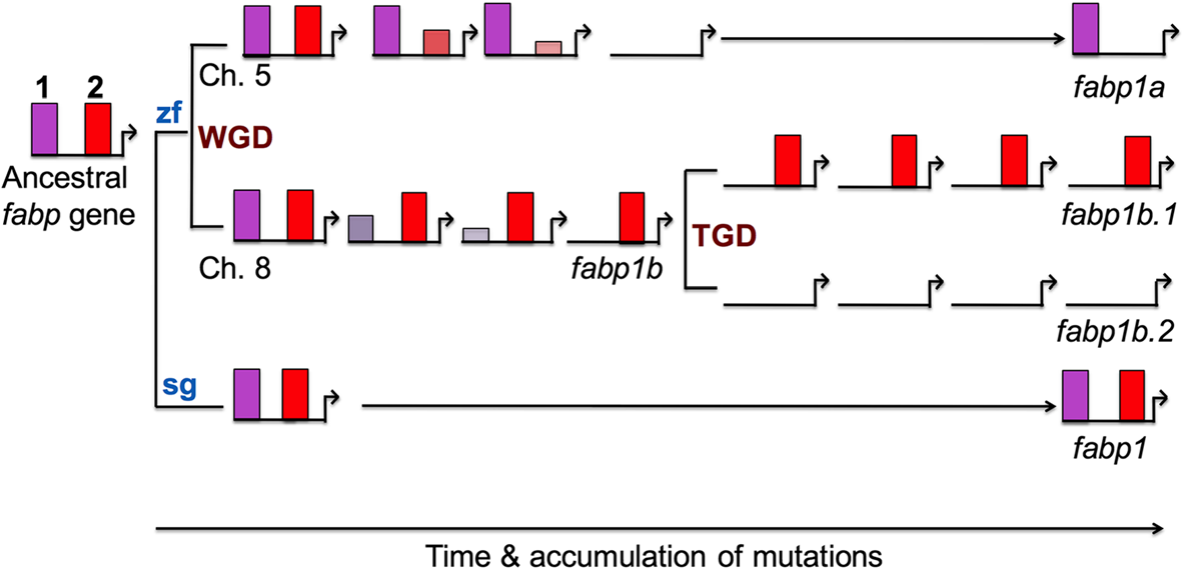
PPAR subfunctionalization of the *fabp1* promoters following the teleost whole genome duplication. An ancestral *fabp1* promoter contained two PPREs that had high sequence identity to the consensus sequence for PPREs. These PPREs were retained in the spotted gar *fabp1* gene promoter. In zebrafish, *fabp1a* and the ancestral *fabp1b* underwent subfunctionalization. *fabp1a* retained the PPARα-selective PPRE and *fabp1b* retained the PPARγ-selective PPRE, following whole genome duplication. Further subfunctionalization of promoter function occurred following the tandem gene duplication of zebrafish *fabp1b* resulting in *fabp1b.1* and *fabp1b.2.* Passage of time and accumulation of mutations is represented from left to right. PPREs are represented by purple (**1**, α) or red (**2**, γ) rectangles. PPRE loss is represented by smaller, more transparent rectangles. zf, zebrafish; sg, spotted gar; WGD, whole genome duplication; TGD, tandem gene duplication

The data reported here raise two questions. First, how did the divergent transcriptional regulation of the zebrafish *fabp1a*, *fabp1b.1*, and *fabp1b.2* genes by PPARs arise? Second, why was this divergent regulation of *fabp1a*, *fabp1b.1*, and *fabp1b.2* by PPARs not selected against? To answer these questions, we must consider the data from this study and how it might be integrated into the existing model of gene duplication, particularly in teleost fishes. The spotted gar *fabp1* gene, used here as a surrogate for the ancestral *fabp1* gene promoter, contained at least two functional PPREs that resembled the zebrafish *fabp1a* and *fabp1b.1* promoter PPREs, respectively, in both their location relative to the TSS, and their PPAR-isoform selectivity. The existing model of gene duplication in teleosts suggests that zebrafish *fabp1a* and ancestral *fabp1b* genes arose by WGD [9, 12, 14], whereas the zebrafish *fabp1b.1* and *fabp1b.2* arose by tandem duplication of the *fabp1b* gene during misaligned unequal crossing over during meiosis [12]. From these data, we can construct a model for divergent regulation of the *fabp1* genes within the context of the DDC model (Fig. 7) [2–4]. The most straightfoward explanation for the retention of *fabp1a, fabp1b.1* and *fabp1b.2* in the zebrafish genome is the ancestral teleost *fabp1* gene was duplicated during a WGD event, which was later followed by a tandem duplication specific to zebrafish. We showed here that increasing concentrations of the PPARα-selective agonist, WY14643, enhanced and then repressed spotted gar *fabp1* promoter activity (Fig. 6). While one might invoke recruitment of a transcriptional repressor of the spotted gar *fabp1* promoter activity, this is not the most parsimonious explanation. Elimination of either PPRE-1 or PPRE-2 abolished the biphasic response of spotted gar *fabp1* promoter activity suggesting PPARα-dependent functional antagonism of the spotted gar *fabp1* promoter activity, which occurred *via* an interaction or competition between the two identified functional PPREs (Fig. 6). No functional antagonism was observed in the zebrafish *fabp1a* and *fabp1b.1* promoters (Fig. 5). We suggest that following the teleost WGD, mutations may have accumulated independently in the PPARα-selective PPRE of the zebrafish *fabp1a* promoter, and in the PPARγ-selective PPRE of the zebrafish *fabp1b* promoter, leading to elimination of these elements in their respective promoters and loss of the functional antagonism observed in the spotted gar *fabp1* promoter (Fig. 7). Zebrafish *fabp1a* and *fabp1b* promoters, therefore, underwent subfunctionalization relative to *fabp1* with regard to PPAR isoform specificity. Subsequent tandem duplication of the zebrafish *fabp1b* gene resulted in a PPARγ-selective PPRE in *fabp1b.1* and the loss of a functional PPRE in the zebrafish *fabp1b.2* promoter with retention of basal promoter activity (Fig. 7) [7]. Zebrafish is unique among teleosts for having three *fabp1* genes [12]. These data demonstrate that *fabp1a* and *fabp1b.1* genes have retained their functional regulation by PPAR, and therefore their association with PPAR-dependent metabolic and hormonal signaling pathways [16]. In contrast, the local (tandem) duplicate *fabp1b.2* is retained in the genome, but its transcription is not modulated by either dietary fatty acids or PPAR agonists. As such, the zebrafish *fabp1b.2* gene does not appear to be associated with PPAR-dependent physiological processes [7, 12, 14]. This conclusion is consistent with cross-species analyses that have shown genes retained from WGD events often belong to signalling networks, whereas local gene duplication events are more likely to acquire network-independent functions [23, 24]. Additional research is required to understand what other regulatory elements have undergone neo-, non-, or subfunctionalization in the zebrafish *fabp1* promoters compared to the spotted gar *fabp1* promoter [3, 4, 25–28]. Our observations provide an example of increasing intra-organismal complexity through the subfunctionalization of response to PPAR stimuli among gene duplicates.

## Conclusions

The present subfunctionalized state of PPAR responsiveness in the zebrafish *fabp1a*, *fabp1b.1*, and *fabp1b.2* promoters may represent a form of segregation avoidance such that three gene products sharing similar function are expressed in different tissues, under different developmental or environment conditions [27, 28]. These data demonstrate the divergent, PPAR isoform-specific regulation of zebrafish *fabp1a*, *fabp1b.1*, and *fabp1b.2* in relation to their subfunctionalization across evolutionary history using a unique pharmacological approach.

## Methods

### Zebrafish and spotted gar *fabp1* promoter sequences

Promoter sequences for zebrafish *fabp1a, fabp1b.1*, and *fabp1b.2* and spotted gar *fabp1* genes were obtained using the CNS Discovery Pipeline (v. 3.0) created and described by Turco et al. [19]. The source code for the CNS Discovery Pipeline 3.0 is available for download at https://github.com/gturco/find_cns with instructions for installation at (https://github.com/gturco/find_cns/blob/master/INSTALL.rst) [19]. CNS Discovery Pipeline was run using default settings except that the filter for promoter regions containing gene-coding regions was removed. The input was the zebrafish Zv9 whole genome assembly (GenBank Assembly ID GCA_000002035.2) and spotted gar Linkage group LG2 LepOcu1 representative genome assembly (GenBank Assembly ID GCA_000242695.1, Gene symbol LOC102694982) [29]. The length of promoter fragments retrieved by the CNS discovery pipeline was determined as the region within 4,000 bp 5′ of the TSS containing > 60 % sequence identity to the consensus of vertebrate transcription factor binding motifs [19]. The resulting “.fasta” output files for the *fabp* promoters and their corresponding genes were used to design PCR primers to clone *fabp* promoter fragments (Additional file 1).

### Identification of putative PPREs in teleost *fabp1* promoters by in silico analysis

Promoter sequences were analyzed for putative PPREs using MatInspector (v. 8.1) with the Genomatix ElDorado genomes database and the vertebrate matrix group. The PPRE was defined as 5′-CAAAACTAGGTCANAGGTCA-3′ [16–18]. The mismatch threshold was set to 35 % (*i.e.* transcription factor sites were identified if they were 65 % similar to the corresponding IUPAC string).

### Cell culture

Primary zebrafish cell culture methods were adapted from Kan et al*.* [30]. Primary explant cell cultures of zebrafish liver and intestine were obtained from adult male fish. Fish were euthanized with tricaine (10 % v/v) and rinsed with 70 % ethanol in sterile phosphate-buffered saline (PBS). The liver and intestine were dissected, rinsed once with PBS, and incubated in 0.25 % trypsin-EDTA (Gibco, Oakville, ON) for 5 min at room temperature. Tissue was suspended in trypsin-EDTA by pipette and centrifuged at 500 x g for 5 min at room temperature. Cells were resuspended in media containing 50 % Leibovitz’s L-15, 35 % high glucose DMEM, 15 % Ham’s F-12, 5 % FBS, 0.15 g/L sodium bicarbonate, 15 mM HEPES, 0.01 mg/mL bovine insulin, and 50 ng/mL human EGF (Gibco) and maintained 28 °C, 100 % atmospheric air on poly-D-lysine-coated cell culture plates. Primary zebrafish cells were maintained for 48 h prior to drug treatment. Media was changed daily. All protocols were in accordance with the guidelines outlined by the Canadian Council on Animal Care. All animal protocols were approved by the Carleton Animal Care Committee at Dalhousie University prior to start of this study.

Human embryonic kidney 293A (HEK293A) cells were obtained from Cedarlane (Burlington, ON). HEK293A cells were maintained at 37 °C, 5 % CO_2_ in DMEM containing 10 % FBS and 10^4^ U/mL Pen/Strep. HEK293A cells express PPARα and γ [31], which was confirmed by sequencing the RT-PCR products (data not shown).

### Cloning of zebrafish *fabp1a*, *fabp1b.1*, and *fabp1b.2*, and spotted gar *fabp1* promoter fragments into the pGL3-basic plasmid

DNA fragments containing the zebrafish *fabp1a*, *fabp1b.1* and *fabp1b.2*, and spotted gar *fabp1* promoter region were amplified from genomic DNA by PCR. Genomic DNA was isolated from frozen liver using the GenElute Genomic DNA Miniprep kit according to the manufacturer’s instructions (Sigma-Aldrich, Oakville, ON). The PCR contained: 2 mM MgCl_2_, 0.5 μM forward and reverse primers (Additional file 2), 0.3 mM dNTPs, 1 U *Taq* DNA polymerase, and 40 ng genomic DNA. PCR conditions were: 95 °C for 10 min; 35 cycles of 95 °C for 30 s, 57 °C for 30 s, 72 °C for 6 min; and 72 °C for 10 min. PCR products were resolved by gel electrophoresis and purified using the GenElute Gel Extraction kit (Sigma-Aldrich). Purified *fabp1a*, *fabp1b.1*, and *fabp1b.2* PCR products were digested with *Mlu*I and *Hind*III according to the manufacturer’s instructions (Fermentas, Burlington, ON). The purified spotted gar *fabp1* PCR product was ligated into pGEM-T easy vector (Fermentas) at 16 °C overnight using T4 DNA ligase according to the manufacturer’s instructions (Invitrogen, Burlington, ON). The spotted gar *fabp1* promoter fragment was excised from pGEM-T by digestion with *Nco*I and *Sac*I according to the manufacturer’s instructions (Fermentas). *fabp1a, fabp1b.1,* and *fabp1b.2* PCR products were ligated into pGL3-Basic (Promega, Madison, WI) at 16 °C overnight using T4 DNA ligase according to the manufacturer’s instructions (Invitrogen). The resulting plasmids (p*fabp1a*, p*fabp1b.1*, p*fabp1b.2*, and p*fabp1*) were propagated in ampicillin-resistant DH5α competent *E. coli* (New England Biolabs, Whitby, ON) and purified using the GenElute Plasmid Midiprep kit (Sigma-Aldrich). The pHRL-TK plasmid was obtained from Promega.

p*fabp1a*, p*fabp1b.1*, and p*fabp1* mutant plasmids were generated by PCR-based site-directed mutagenesis. The p*fabp1a* PPRE 5′FR at −2,710 bp was mutated from 5′-CAAAAC-3′ to 5′-TGGGGT-3′ and the PPRE DR1 at −2,710 bp was mutated from 5′-ACAAGT-3′ to 5′-CGTGGA-3′. The p*fabp1b.1* PPRE 5′FR at −1,232 bp was mutated from 5′-CTAAAC-3′ to 5′-TCGGGT-3′ and the PPRE DR1 at −1,232 bp was mutated from 5′-TAAGGT-3′ to 5′-CGGAAC-3′. The p*fabp1* PPRE-1 (−1,953 bp) was mutated from 5′-CCCTA-3′ to 5′-TTTCG-3′ and PPRE-2 (−539 bp) was mutated from 5′-AGGACA-3′ to 5′-TAAAGT-3′. Reactions were composed of 2 mM MgCl_2_, 0.5 μM forward and reverse mutagenic primers (Additional file 2), 0.3 mM dNTPs, 1 U *Taq* DNA polymerase, and 40 ng plasmid DNA. PCR conditions were: 95 °C 1 min, 18 cycles of 95 °C 50 s, 60 °C 1 min, and 68 °C 8 min, followed by a final extension at 68 °C for 10 min. Input plasmid was removed by digestion with the methylation-insensitive *Dpn*I (5 U) in 1X FastDigest Green Buffer® in a final volume of 20 μL (Fermentas, Burlington, ON) for 1 h at 37 °C. The constructs of wild-type and mutagenized zebrafish and spotted gar promoters was confirmed by DNA sequencing of the promoter-reporter gene constructs prior to transfection of HEK293A cells (data not shown).

### Transfection, PPAR agonist and antagonist treatment, and the dual luciferase assay

Transfections of HEK293A cells was performed using lipofectamine 2000 reagent according to the manufacturer’s instructions (Invitrogen) with 400 ng of p*fabp1a*, p*fabp1b.1*, p*fabp1b.2*, *pfabp1,* or pGL3-Basic (background control), and 200 ng pHRL-TK. The luciferase activity of the pHRL-TK plasmid containing the *Renilla* luciferase gene under the regulation of the cytomegalovirus thymidine kinase (TK) promoter was used to normalize firefly luciferase activity under the regulation of zebrafish promoters. Luciferase activity was quantified according to the manufacturer’s instructions (Promega).

HEK293A and primary zebrafish cells were treated with rosiglitazone (PPARγ agonist), WY14643 (PPARα agonist), T0070907 (PPARγ antagonist), GW 6471 (PPARα antagonist), or vehicle (0.5 % DMSO) at the concentrations and times indicated [32, 33]. All PPAR agonists and antagonists were purchased from Sigma-Aldrich.

### Quantitative reverse transcriptase PCR

RNA was extracted from HEK239 cells using Trizol® (Invitrogen). Reverse transcription reactions were carried out with SuperScript III® reverse transcriptase (+RT; Invitrogen), or without (−RT) as a negative control for use in subsequent PCR experiments according to the manufacturer’s instructions. Two micrograms of RNA were used per RT reaction. qRT-PCR was conducted using the LightCycler® system and software (version 3.0; Roche, Laval, QC). Reactions were composed of a primer-specific concentration of MgCl_2_ (Additional file 2), 0.5 μM each of forward and reverse primers (Additional file 2), 2 μL of LightCycler® FastStart Reaction Mix SYBR Green I, and 2 μL cDNA to a final volume of 20 μL with dH_2_O (Roche). The PCR program was: 95 °C for 10 min, 50 cycles of 95 °C 10 s, a primer-specific annealing temperature (Additional file 2) for 5 s, and 72 °C for 10 s. Experiments always included sample-matched –RT controls, a no-sample dH_2_O control, and a standard control containing product-specific cDNA of a known concentration. cDNA abundance was calculated using the ^ΔΔ^CT method and normalized to GAPDH levels [34].

### Statistical analyses

CRCs were fit using non-linear regression analyses [variable slope (four parameters) and Bell-shaped] in GraphPad Prism (v. 5.0). Statistical analyses were conducted by one-way ANOVA followed by Tukey’s *post-hoc* test or two-way ANOVA follow by Bonferroni’s *post-hoc* test, as indicated. Homogeneity of variance was confirmed using Bartlett’s test. All results are reported as the mean ± standard deviation (SD) or standard error of the mean (SEM), as indicated, from at least three independent experiments.

## Abbreviations

5′FR, 5′ flanking region; CNS, conserved non-coding sequence; CRC, concentration-response curve; DDC, duplication-degeneration-complementation; DR1, direct repeat region; fabp, fatty acid-binding protein; HEK, human embryonic kidney cell line; PBS, phosphate-buffered saline; PPAR, peroxisome proliferator-activated receptor; PPRE, peroxisome proliferator-activated receptor response element; RT, reverse transcriptase; SEM, standard error of the mean; SD, standard deviation; sg, spotted gar; TGD, tandem gene duplication; TK, thymidine kinase; TSS, transcription start site; WGD, whole genome duplication; zf, zebrafish

## Additional files

Additional file 1: Conserved non-coding sequence files (fasta) analyzed in this study. Putative PPREs are indicated as green 65–74.9 %, yellow 75–84.9 %, orange > 85 % sequence similarly to the defined PPRE consensus sequence. Red indicates primer binding sites for PCR. Light blue indicates regions of repetitive TAT sequences. DNA sequences determined by sequencing PCR-cloned promoter fragments of spotted gar or zebrafish genomic DNA. (PDF 38 kb) Additional file 2: Table S1. Synthetic oligonucleotides used for cloning *fabp1* (spotted gar), *fabp1a* (zebrafish), *fabp1b.1* (zebrafish), and *fabp1b.2* (zebrafish) promoter fragments, mutagenesis of the *fabp1*, *fabp1a*, and *fabp1b.1* PPREs, and quantification of *fabp1a*, *fabp1b.1*, and *fabp1b.2* transcripts *via* qRT-PCR in this study. (PDF 82 kb)
