# Supplementary material for: Subfunctionalization of peroxisome proliferator response elements accounts for retention of duplicated fabp1 genes in zebrafish
Source: BMC Evol Biol. 2016 Jul 16;16:147. doi: 10.1186/s12862-016-0717-x (PMC4947323; doi:10.1186/s12862-016-0717-x)
Supplement: Additional file 1: — Conserved non-coding sequence files (fasta) analyzed in this study. Putative PPREs are indicated as green 65–74.9 %, yellow 75–84.9 %, orange > 85 % sequence similarly to the defined PPRE consensus sequence. Red indicates primer binding sites for PCR. Light blue indicates regions of repetitive TAT sequences. DNA sequences determined by sequencing PCR-cloned promoter fragments of spotted gar or zebrafish genomic DNA. (PDF 38 kb) [file 12862_2016_717_MOESM1_ESM.pdf]

Appendix S1. Conserved non-coding sequence files (fasta) analyzed in this study. Putative PPREs are indicated as green 65 – 74.9%, yellow 75 – 84.9%, orange > 85% sequence similarly to the defined PPRE consensus sequence. Red indicates primer binding sites for PCR. Light blue indicates regions of repetitive TAT sequences. DNA sequences determined by sequencing PCR-cloned promoter fragments of spotted gar or zebrafish genomic DNA.

>Spotted gar (*Lepisosteus oculatus*) *fabp1* promoter
ACACTGGTCACTCTGGTCAATATGCACACAATAGAAAACACACATTACACACTAGTCAACACAC
ATTACAAACTGGACTGCCCACTGGTCAACACACATAACATACTGGACAACACTCTGGTCAACAT
GCATGACACACCACATGTGAGCAAAAGGCCAGCAAAAGGCCAGGAACCGTAAAAAGGCCGCGTT
GCTGGCGTTTTTCCATAGGCTCCGCCCCCCTGACGAGCATCACAAAAATCGACGCTCAAGTCAG
AGGTGGCGAAACCCGACAGGACTATAAAGATACCAGGCGTTTCCCCCTGGAAGCTCCCTCGTGC
GCTCTCCTGTTCCGACCCTGCCGCTTACCGGATACCTGTCCGCCTTTCTCCCTTCGGGAAGCGT
GGCGCTTTCTCATAGCTCACGCTGTANGTATCTAGTTCGGTGTANGTCGTTCGCTCCAAGCTGG
GCTGTGTGCACGAACCCCCCGTTCAGCCCGACCGCTGCGCCTTATCCGGTAACTATCGTCTTGA
GTCCAACCCGGTAAGACACGACTTATCGCCACTGGCAGCAGCCACTGGTAACAGGATTAGCAGA
GCGAGGTATGTAGGNGGTGCTACAGAGTTCTTGAAGTGNGGCCTAACTANNCTACACTAGANAC
AGTATTTNGTATCTGCGCTCTGCTGAGCCAGTTACCTTCNGAAAAANAGTNNNAGCTCTNATCC
GGCAACAACNNNCTGNAGCGGNGGNTTTTTTTGNTTGCAGCAGCANAANTACNNNNNNNNNNAA
AAAAAGGNNNNNNNNCAANNAANNANTCNTTTGNANNNNTTTTNNNNNNCGAGAGAAACGGTAT
CTGTAGAGTTTTCTCACACGCAAGAAGGCACACAGTCCGCACTTACAGGCTCGTCGCTGCGGTT
ACTGTGGACTTTCTACGCACAGCACTGCGAATGCAGCTTCAGCCTGTTACAGACCGCGGCTATT
ATTAGGACTGGCGTCACCAGCGAGACTGCGTGTCCTGCCGTCCGCACCGCGGTCACTTCGGCCG
AGCCGAGCGCTGGACACGGCGCAGTGGGCGGCCCGGGTCCCGGAGCGGGTCTCGGGGCAGAGTC
CACGCCTGCTCTTCCACACACTCCGCCCACAGGGCACGGGGCACGGGGCACGGGGCGAGGGAAC
CCGCTGCCTGCCGGACAGCGCCCTCTGCTGCCGACACGCAGACACACGGCCAGCTCTGACAGCA
GAGCGGGAACCTCAGTCTCTCCCTCTTCTGATCATTGTACACACCATTCTAAATGCAGCCTGAC
ATCTTCGACACGACCAGCTTTGATTCAAAACTTTTCCCTAGGGTTCCCTCACTTTTCACTAGAT
ATCCTGGTATTTTTATTGCTTCCCACATTGCCTGGGAGACGCAGGCTAAGCGATGTGTCGACAT
AGTCACCCAGGTCTTTTTCACAAGAACTGTATCATCTTCTAGCTCAATATTTCTCACTTTGTAT
TTACAGTTATTTTGTCTACCTGAACCCTACCAAAACCCTGCATCTGTCTGTATCGAGGGCTGAT
GTTTCACTGGTTTTCTCAGCTGTGATACTGTGCTCTGTGTCCGTTCCTCAGAGGCGCCAGTCTT
GTAAATGATTCACTTCTTGCTCGTTTTTCTTCTTACATACAGAAATCTGATCATGATCAATAAA
CTCAGACACATTAAGCCTTTCTGATGAGCATCTCTTGTATCACATTTACAGTATTAGGGTCACA
AGTAACAGGAGTGAGTCAGTCTGGACTCTGCTCCCTGGAGACACGTCTGAACTCCTGTTGGGAC
ACAGACACATCACTGTGTGTGTCTGTTTATCTGTGCTGCTGCAGGCCAGGTGGACTGAATTTAT
GGGCAGCAATGCTGTCTCGCAGTGCTGGGGCCCTGGGCTCAGTCCTAGACCTGGGGCGTTTTCT
CTCTGGAGTTCATCTGCTCTCCCTGTGTTTGCGTGGGTTTCAATCAAATTCTCTTTCTGGTTCG
CTTTCTATTCAAGTAGCTCCACATGAGGTGAAAACAGACCAGAAACAAATTGTTTAGGCTCCTT
TGGATTTAAAGAAGATGCCTTCTGATTTCTAAGAAAATATGACATCCTTACATCACCTGTATGT
CAGGAGCTACTGTTATTTCTGGGGACGTTTTCTCACAGTTTGTGCTATTTGCGCTGCATGAGTG
ATGCTAAAACACACACACCTTCGCTCAGACACACCCTGTACCTGGTGACTCCCTGCACTGATAA
CAGCCCAGCAGGTAACCTGTGGTCACACTGATCTTTGCTTTGACTGCAGAACTCTTGCTGTCTG
TGACTTTTGTAACTTCTGTAACAGTGCGAGTTATCAGGTGTCTCTGCGCACATTGGACAGCCTA

GACCTCCACAGCCCCAGCTCTGTACAGCAGAGAAACCTTTACTCTCCCACTGCACAGCACTGAC
AATTCAGACACTGCTCTGACTTTTCGTCCATATGTTTTATCATTCCTAACAACTTGAAACAAAT
TGTGCTCTGGATTAAAAAAAAATTAAAAACTGAATTCTAAAAAAGGTTATGCAATTTTGTTATT
AAATACTTAAAGGGTTTACATGGTGCAATGCATGGGGTATACAGTATACTGCAGTGCAATAGAG
AAGTCTATAAACTGTAGTACAGGAGTCTGTGTAGTGTAGTACAGGAGTCCATAAACTGTAGTAC
AGGAGTTGATATAGTGTAGTACAGGAGTCTATAAACTGTAGGACAGGAGTCTGTGTAGTGTAGT
ACAGGAGTCTGTGTAGTGTAGTGCAGGAGTCTATAAACTGTAGTACAGGAATTTATATAGTGTA
GTACAGGAGTCTATAAACTGTATTACAGGAGTTAATATAGTGTAGTACAGGAGTCTGTGTAGTG
TAGTACAGGAGTTTATATAGTGTAGTACAGGAGTCTATAAACTGTATTACAGGAGTTGAAATAG
TGTAGTACAGGAGTCTGTGTAGTGTAGTACAGGAGTTTATATAGTGTACCACAGGAGTTTATAT
AGGGCTGTACCCCAATCAGACTGGTGTATCCCCATGATCGCTGTGTTTTGATGTAATTCCGAAA
ACAGCAGACAGAGAGATCCTTTAAGTCCCGGAGCTGCCGACACCAGCGCTGAGAGGCTCCACTC
CTTGGCCCTTATCTTATCTGATCAGGGCTAGAGGTGGGCCTGCCGCGGTCCTCAGCGGGCCTGA
GGTCATCCGACCGCCCCGGCGCGCCGCCCCCCCGCCCTATAAACCACCCGGTCCCTGCGCGCTC
CCGCCGCCCGACCGCAGCG

>Zebrafish (*Danio rerio*) *fabp1a* promoter
TAAGTTTCTGCAATCCCGGAAAGGAACCGACTCATGCGACTGTCATGTGATGTGCGTTACTTTT
CGCAGCGCTGCTGTGAAGAGCCAATCACAACTGTTTCTGATTATTTACATATTTTTACGCAAAA
TAAGAATCATTCTTCATTAAACACAAACCCTAATACTGTTACAATACCAACAGCATAAAATCAT
ATTTATAATAAAAAAATAACACAATTTTATAGACCTACAAGCCTGTGATCAAGTGATGAGTCGA
GTAACGTAGATAAATTAAATTTAAATGAGGCAAAATAACACCGAAGCTCGATAGTAGCCTGTGA
TCAAGTGATGATAACAAGATATTATTAATTTCTTTGAGACAATGATATTTTTATGGTAGCTCAT
GAATCTGCAATCCCGGAAAGGACATACGAGATCTTTTATTAAACCTTATTTATATATTTTTTCC
CTCAATGTTTTGATTGTCAATACCTTCGGGATATAAACATAAAGATGTGTTTAACTTCCAATTA
GACAAACGTTTCCAAACCTTGGATACAAGTAATTCAATTATTATCTTATACAATTGTAATACAA
TTATTGAAAGTATATGAGTGTGTAAAGATATAATTAAACCAAATGCAGGAGTGTCAATGCAGTG
CACATTGAAAATACCAATAAAAGCACATCTTCCTTCAGCTTAGTCCCTTTATTCATCAGGGGTC
GCCACAGTGGAGTGAACCGCCAACTATTCCAGCATGTGTTTTACACAGCGGATTCCCTTCCAGC
TGCAACCCAGTACTGGGAAACACCCATACACTCTCATTCACACACACTCATACACTACGGCCAG
TGTAGTTGATCAGTTCCCCTATAGCGCATGTGTTTGGACTGTGGGGGAAACCGGAGCACCCGGA
GGAAACCCACGCCAACACGGGGAGAACATGCAAACTCCACACAGAAACACCAACTGACCCAGCC
GACACTCAAACCAGCGACCTTTTTGCTGTGAGGCAACAGTGCTAACCACTGAGTCACCGTGCTG
AACGGTCACCTGTTTTTGAGATTAAAAATAAACACATACAAACAAATCTAAATCAATTCCTGTG
GTCTTTTTAATTACTTCATAAATCCATAAACAAAACCTGGATACAAGTCAAAAGGAGTTATTAA
AACTAATATGTGTTGAAATAAAGGCGTATTTATGTATTTATATATCTATTTATTTATATATCTA
TCTATATATGTATTTATTTATTATTTATGGTATCGTATNNNNNNNNNNNNNNNNNNNNNNNNNTA
GTGTTCATGGCAGTCAGTGTGAGCTTCCAGTTCCAGAAAGATTTTTTTAAATCTTCCATTAAA
ATAAAAAAGAAAAAGGAAAGTAAAAAAAACATGGATGAGTTTGTATGAGTAAAATTACAACAAA
CATTTATTTTTGAAAAACAGGGAATTTTGCAATAGTTCATGCATTCTCATGTTGTTGTAAACTT
ATATGACTCGCGTCTCTAAATCACAAATGGGAAATTCTGAAGAATCATTACAGATCTCATTTCA
AAACAAGAACATTTCATAAATATCTTGATGTCAATATCAAGCAATAAATGCCACCCACTAAATT
CCAAGTGATTTTTAATCACTACTTTTTTTGCATGTACATCTCAAATATAAGTATTTCCGTCTGC
TAGAGCACTGTTCATGCTTTATTTGTCGAGTATTGGGGGTGTTATGTGAACAAAAACCTCTTTT
AGGTCTTTAGAATAGAATTTCTTTCTCTCTTTGTACGTTTATAAGTTTGTGGTGTATATATATA
CTTTGATGAGAGTTTGACATCTTGCTCTACAATATGATCTGCTAACACATGCTAAAATGTGCAT
TTTGAACAATAATTAAATCAGCAGCACGGTGGCGCAGTGGGTAGCACAATCACCTCACAGCAGG

AAGGTCACTGGTCCAAGCCTCGGCAGGGTCAGCTGGTGTTTCTGTGTGGAGTTTGCATGTTCTC
CCCGTGTTGGTGTGGGTTTCCTCCGGGTGCTCTGGTTTCCCCTACAAGTCCAAAGACATGCGCT
ATAGGTGAATCAAACTAAATCAGGGTTTCTGCAGGTTTCTCAGAGTTATATGTAAGACTTTTCA
GACATTTTTAAGACCTTAATGAATTAATTTTTAGACCCATAAAGGGCTAAATGCAAAAGATTTT
TTTAATAGCCCAGATGGAAAAGATTTTATTTGCCCTATCAATTTACACTTAATTATAATACTTT
TAATAATACAATAATAATTTAATAATACAATTTTTGCATTATTTTAGATTTTATTTCTTAGCAA
AATATTTTGCATATTGTGTGAAATCAAGCAAGCTCTACATGTTCCCATACACTTTTATTCCAAC
ATAAACTTAAAGAAAAATAAACAAACAAAAGTTTTAAAAACTATAATTAACTAAATGTATGCAC
AACAATCAGTCCAGGTCAGTATCCTCAGCAGTAAATACATGGAGCACTTTTAAACAAATGTAAT
AGTAAGGAAAATTATTTATCTTAAAAGTAAAATATTGCATTAATTTAAATAAAAGGTTTAATGT
TTTATTGAGAGGATTGTGGGTGATTGTATGGGTTTTGGTCAGATTTGGTAGCAATACATGAACA
AAGAAAAATTAAGACCTGTTTAAAACAGATTTAAGATCTCCAACACAATATTTCAGAACATTTA
AGACTTTTTAAGGCCTAAAATTTAGATTTTGAGATTTAAGACATTTTAAGACCCTGCAGAAACC
CTGTAAACTGGCCGAAATGTACGAGTATGTGTGTGATTGAGTGTGTATGGGTGTTTCCCAGTAC
TGGGTTGCAGCTAAAAAGGCATCCGCTTAGTAAAACGCATGCTAGAATATTTGGTGGTTCACTC
AGCTGTGGCGATCCCTGATGAATAAAGGGACTCAGCTGAAGGAATATGAATGAATGAATCTTGC
AAAACATCTGACATAATATTACAGTATATACTGCCATACGGCATAATAAATCAGTTATTAGAAA
CAAGCTATTAAAACCAGTATGTGTTGAAAAACATCTTCTCATAAATAAACAGCACTTGGGAATT
ATTTGAAACAAAAAATAATAATTAAAATGAATATACGTGAGAGTCGTGAGAAAGCGGAAACGTC
TTGAGTTGTGTGCAACCTTTGGACATTTATTCCAGACTGGCGCATTTTCTCAGTGGCATAATCA
TAATCTGATCTGTCTTTATATAACGGTGTATTGTGCATTGATAGGTTTAAAGTCTGACGTAACA
AACCCTCAAGGAGAAGAAAACCGCCTGTAGGAGCAAAACGCTGC

>Zebrafish (*Danio rerio*) *fabp1b.1* promoter
AATCTGTTGTGGTGATTCTACAGTTGCTATGGTAACACGTCAACTATAGTAATTGATCTATTGT
GGTGATTCTACAGTTGCTATGGTAACACGACAACTATAGTAAATGATGTGTTGTGGCAAGTCTA
CAGTTGCTATAGTAACACAACAACTATAGTAAATAATTTGTTGTGGTGATTCTACAGTTGCTAT
GGTAGCATAACTACTATAGTAATGGATCTATTGTGGTGATTCTACAGTTGCTATGGTAACACGA
CAACTACAGTAAATAATGTGTTGTGGTGACTCACAAAAACTAGATTAAAGACCTATCTGTTTAG
TAAAGCATACACTCAGTGCATCACCTAGCAGGTTCCACACTGGCTTCTGCATCTTGCTTATATA
CACTATGAACAGCAGCTACGCTAATTATTCTCTTTATTCTCTATTTTCACCTGGGGATACTCAT
CCCGAGGTCCTCAGATTAGGCGGAGTCACTGATTGGATCCAAGACCAGCGACGTGATGATCCCA
AGGATTCCATATCCGGGACCAGGCCATATCCTGAGCTGCTGCTGCGCTGATGGTCGTGGGGAGT
GGAGAACATGAGTCTGATTCCAGCGACGCTCCAGGGACAGACGAGTCTTCATTGAGGCCATCTT
CCAGCATAAACCACGGCGAATGAAGTTCTGCACAAGACTTTTGGCCAGCGGAGAAATTAAAATG
GTCGCGCCCAACTGAGTCTGGTTCTCTCAAGGTTTTTTTTCTTCACTCCCATCAGGTGAAGTTT
TTTTTCTCTCTCCGCTGTCGCCACTGCCTCGCATGGTTCAGGATTGGTAGAGCTACGCATCGAT
GAATTTGCTCTTCAGTGTTTGAACTCTCAGTAATGATTAAATCACACTGAACTGAGCTAAACTG
AACTGAACTGAACTTAAACACTAAAACCTGAACCACACTGATCCAGTTACTATGACCATTTATG
TGAAGCTGCTTTGACACAATCTACATTGTAAAAGCGCTATACAAATAAAGCTGAATTGAATTGA
ATTGAATTGAATTGACTCTACAGTTGCTATGGTAACACATCAACTGTAGTAATGGATCTATTGT
GGTGATTCTACAGTTGCTATGGTAACACGACAACTAGAGTTATGGATCTGTTGTGGTGATTCTA
CAGTTGCTATGGTAACACGACAACTATAGTAATCGATCTGTTGCGGTGATTCTACTGTTGCTAT
GGTAACACGACAACTATAGTAATATAAACAAATGAATGAAGTTTTTTCCAACTATAGGGTGTAT
ATACACCACAGTTTACTGTAGTAAACACAGCTTATCATGGTAGTTAATACTACAGTATAAGCTG
GAGCCTTCATTAAGATGTAGATACTTAAGTGAGCGACCACTCATCAGATGTGTGGTTTTTTCCA
CTGGTCTTACATGATTAGTCACACATGTCGCTTTTTCCGAGGCTTCTGTGGCTCTCTGAAGGCC

TCTTTTCTGCTTGGACATGCTCAGATGATGCGGCTCATGAAAATGACACACAGAAAACGGATGT
ATTTACATCATCTCATGACTGATTCATAATTGGTAAGTGAGCGACCAATATAAGCAAGTAGATA
TAAATCAATCAGCACAGCAAAACCTCATGCAGGATGATTCATCCCGGCTGTTTGAAGTGCATGA
CATTCAAATCAGAGCTTGGCTATAAATATGATTGAATGTGTGTGCTATTTACGTCCTGACATAA
CCTTTATCCTGGGCCACGAAGTGGCACAGTAGGTAGTACTGTCGCCTCACATGAAAAAGGTCGC
TGGTTCGAGTCTAAACTACGGATAAGGTCATGAATTGGGTAGGCTAAAATGTCCGTAGTGTATT
ATTATTATTATTATTATTATTATTATTATTATTATTATTATTATTATTATTATTATTATTATTA
TTATTATTATTATTATTATTATTATTATTATTATTATTATTATTATTATTATTATTATTATTAT
TATTATTATTATTATTATTATTATTATTATTATTATTATTATATATGAAAATGTATATGCATACACA
CGGTATACACCCACCGGCCACTTTATTAGGTACACCTGTCCAACACAAATTCCACTTTTGGACG
TGTTTTGGCAGAAGCTCAATGCATTTATGCATGTAGACATGGTCAAGACGATCTGCTGCAGCTT
AAAGTGAGCATCAGAATGGAGAAGAAAGGGGATTTAAGTGACTTTGATCGTGGCATGGTTGTTG
CTGCCAGACGGGCTGCTCTGAGTATTTCAGAAACTGCTGATCTGCAGGGATTTTCACGCACAAC
CATCTCTAGGGTTTACAGAGAATGCTCCGACAAAGAGGAAATCTGCAGTGAGCGGCAGTTCTGT
GGGCGCAAATGCCTTGTTGATGAGGCCAGAGGTCAGAGGAGAATGGCCAGACTGGTTCCAGCTG
ATAGAAAGGCAACAGTAACTCAAATAAGCACTCGTTACAACCGAGCTCTGCAGAAGAGCATCTC
TGAACACACAACACGTTCAACCTTGAGGCGGATGGGCTACAGCAGCAGAAGAGCACACCGGGTG
CCGCTCCTGTCAGCTAAGAACAGGAAACTGAGGCTACAATTCACACAGACTCACCAAAACTGGA
CAATAGAAGATTGGAGAAACGCTGCCTGCTCTGATGAGTCTACATTTCTGCTCACACATTCAGA
TGGTCGGGTCAGAATTTGGCCTCAACAACATGAAAGCATGGATCATCCTGCCTTGTATCAGCGG
TTCAGGCTGGTGGTGGTGGTGTAATGGTGTGGGGGAGATTTTCTTTGGCTCCATTAGTACCAAT
TGAGCATCAACGCCACAGCCTACCTGAGTATTGCTGCTGACCATGTCCATCCCTTTATGAGCAC
AGTGTCTCCATCTTCTGATGGCTACTTCCAGCAGGATAACGCAGCATGTCATATCCAACTTTCT
ATTAGTCCAGGTGTTTCCCAGTGTTGGGANNNNNNNNNNNNNNNNNNNNNNNNNNNNNNNNNNN
NNNNNNNNNNNNNNNNNNNNNNNNNNNNNNNNNNNNNNNNNNNNNNNNNNNNT

>Zebrafish (*Danio rerio*) *fabp1b.2* promoter
TGTGTGTGTGTGTGTGTGTGTGTGTGTGTGTGTGTGTGTGTGTGTGTGTGTGTGTGTGTGTGTG
TGTGGGTGTGTGTGTGTGTGTGTGTGTGTGTGTGTGTGTGTGTGTGTGTGTGTTTGTGGGTACACT
GCAAAAAATGCTTTTCTTACTTAGATTTTTTGTCTTGTTTCGAGTCCAAATATCTAAAAACAAC
ATGCTGGAACGGGAATGTGTTTGTGTGTATTATTTATTTATTTATGATTAGTAATATTATTTAT
TTATTATTATTATTTATGTATTATTATTATTATTTAATTATATATTATTATTATTATTATTTAT
GTATTATTATTATTAATAATAATATTATCTATTTATGTATTATTATTATTATTATCATTATTAT
GTATTATTATTATTATTATTATTATTATTATTTATGTATTATAATTATTTATTTACTAATTATT
ATTATTATTAGTAGTAGTAGTAGTAGTGGTATTAATAATAATAATAATAATAATAATAATAATA
ATAATTTATTAATATTAATATTATTCATTTATTTATGATTAGTATTATTCATTTATTTATTTAT
TTATTTATTATTTATGTATTATTAGTAGTAGTATTAACATTATTTATGTATTTATTTATTTATT
TATTTATTACAATTATTATGGCTGCACGGTGGCTCAGTGGTTAGCACTGTGGCCTCACAGCAAG
ATGGTCACTGGTTCGAGTCCTGGCTGGGTCAGTTGGTGTTTCTGTGTGGAGTTTGCATGTTCTC
CCCGTGTTGGTGTGGGTTTCCTCCAGGTGCTCCAGTTTCCCCCACAGTCCAAACACATGCGCTA
TAGGGGAACTGATCAACTAAACTGGCCGTAGTGTATGAGTGTGAACGAGTATGGGTATTGTATG
GGTGTTTCCCAGTGTTGAGAAACCAGAGCACCCAAAGGAAACCCACGCCAACACGAGGAGAACA
GTTAGGTTTGACCCAATGTTAGGGTTATGGCAATGCAGCATGTTTTAGATTAATGATAAGTGTG
TGTGCGTGTGTGTATGTGTGTGTGTGTGTGTGTGTGTGTGTGTGTGTGTGTCAGTAGTAGTAAT
GCCAGTCTGATGTTAAAATAGTTTTATAACCGCTGGGTGAAAATTGACCCAAAGGACATTTATT
CATTTTCTTTTCTGCTTAGTCCCTTTATTAATTTGGGGTCGCCACCGCGGAATGAACCGCCAAC
TTATCCAGCATATGTTTTTACACAGTGGATGCTCTTGTAGTGTCGTAAATTTTGACGTCTCATC

AATTACAAATTATCAGACCGGAGGTTTTGAATATCAGAAAATAGACTTTATTCTCCATAATAAA
GCCGAGAAAGAGCAGAGCAGACCCCAGAGCACTCTGAAGTCTCTCCTGGACACCTTCTAAAGTC
TCTCCTGCTCCTTCCCCCTTATTTATGTTTTTGACACACCCCTATGTGTCTCTTTTTAACTTTT
GATCATTTTTGAATAGGTTTTCTAGTCTAAGGGGTCCTGCTATTCTTGGCTCTCAGCCCATTAG
CTCATGTCAAACAGAGATGTTACAGGTCTGTGTCAGCAAGGTATAGATGCAACTTATGCAAAAG
AACTAAGTGTTTACATACATTGTTATGATAGGATAATGACTTGATAATATGTTACTCATTCTAA
CTTCTGACACATATAGCTTCATAGAAGTATTTAACATATATAATCAGTGCTTTACATATTCAGT
TATTCTACACAATCAGTCACTCAGCCTTCTCCTAGTGTTGCTCCTGTGCAGGCGTACTCATCTT
ACACAGACATATCACTGTATTTTTAACACAGGCTGGCATGTTTGCTGCAAACACTACATACATT
TTGTGAAGAGAAAATTAACTGCTTTACACTTAGAAAATACTTCATACAGAGAGAATAAAATCTT
CTTCACTAGCTGCAACCCATCTCTGGGAAACATCTATACACACTCATTCCCACATTGTTTACAT
CCTAAACGCTGGACACTGTGCTGGTTATTGATCGATGTGTAAAAATAAGAATACTCACAGGTTG
TGTTTCACAATCCACAGCTTCATCTATTCTGGTTGAAATCGTCCATTTGAGGAGTTTTCAGCCC
AATCCAGCTCTGAAGTGGAAGAGATTCTGGAAGAGTTTGGCAAAGCTGGTTTATGTTAGCACAT
TCAGACTTTCACGTTCAATAAATTTGTTAATATTAATAATAATAATAATAATTCCTTACATTTA
TACAGCGCTTTTCTGGGCACTCAAAGCGCTTTACACATGGGGGGAATCTCCTCATCCACCAATA
GTGTGCAGCATCCACCTGGATGACGCAACGGCAGCCATAGACTGGAGGAACTATGACGTCACTT
TGTAGGCTAATCGGACGCTAGCATAAAACTGGTTCCCTCGTGAAAATCCCTATAGGATTTTCCC
ATAGGATTTTCGAAGATTGCACATAATAAGCTCTGTGTTCAAACACAGTTCATTACACTTACAC
GTTTTGTCCAGCCGGATAATCCTCACACGAAAATACAACTTTGAGCACTTTTGGATCTTAAATA
CAAACGCAAGAACTGAAAAGCTAACATTAGGCTATAAACGGACTACAGTGCCCTCGGGGCGCTC
AGCTGTGACGTCAGCCCCACCCTGCTACAGACGACTTATCAAGCTTATTTTTAGAAATATAGTC
CATGACAAAGAGTTGATCTCTCTGTTTATTTATTATAATCCACATTATATATTATAAACAAATA
AATACGCTAAAACACATAGACATATGTGCCTTCTGGATCGTATAATATATATGTGTGGAGTTTG
CATGTTCTCCATGTGTTTGTATAAGAGTGTATGAGTGTTTCCCAGAGATGGGTTGCACTGGGAA
GGGCGTCCGCTGCGTAAAACATATGCTGGATAAGTTGTCGGTTCATTCCGCTGTGGCGACATCA
GAATAATAAAGGGACTAAGCTGAAAAGAAAATGAATGAAAATATTCTCAGTATTTCGAAAAAAG
CTCAAAGGGCATCAGTAGTTGTGAGAATCCTGACACACAGCGTTCTCTCACACACACACACTGA
TTTGTTTAGTCCTCCAGCAGTGCTGAATTTGCCTGTTGATGAGTTGCCTGAGCGAGCCTCATTA
AAACATGCAAATTCAGCTCCAGGGGGCCAATCAGAAGCCTGCATTCTGGTGTATAGCTGTTACT
CATTGATGTTGAGTGTAT
