# Supplementary material for: Subfunctionalization of peroxisome proliferator response elements accounts for retention of duplicated fabp1 genes in zebrafish
Source: BMC Evol Biol. 2016 Jul 16;16:147. doi: 10.1186/s12862-016-0717-x (PMC4947323; doi:10.1186/s12862-016-0717-x)
Supplement: Additional file 2: Table S1. — Synthetic oligonucleotides used for cloning fabp1 (spotted gar), fabp1a (zebrafish), fabp1b.1 (zebrafish), and fabp1b.2 (zebrafish) promoter fragments, mutagenesis of the fabp1, fabp1a, and fabp1b.1 PPREs, and quantification of fabp1a, fabp1b.1, and fabp1b.2 transcripts via qRT-PCR in this study. (PDF 82 kb) [file 12862_2016_717_MOESM2_ESM.pdf]

Table S1. Synthetic oligonucleotides used for cloning *fabp1* (spotted gar), *fabp1a* (zebrafish), *fabp1b.1* (zebrafish), and *fabp1b.2* (zebrafish) promoter fragments, n *fabp1*, *fabp1a*, and *fabp1b.1* PPREs, and quantification of *fabp1a*, *fabp1b.1*, and *fabp1b.2* transcripts *via* qRT-PCR in this study.

Target	Sequence (5' - 3')†	Product Length (bp)	[$MgCl_2$] (mM)
fabp1	GGTCAACATGCATGACACAC<br>AGATAAGGGCCAAGGAGTGGA	3,031	2
fabp1a	**ACGGCT**GTTTCTGCAATCCCGGAAAGG<br>**AAGCTT**GTTTTGCTCCTACAGGCGGT	3,300	2
fabp1b.1	**ACGCGT**CCAGGCCATATCCTGAGCTG<br>**AAGCTT**TCCCAACACTGGGAAACACC	2,443	2
fabp1b.2	**ACGCGT**ACAACATGCTGGAACGGGAA<br>**AAGCTT**TCTCACAACTACTGATGCCCTT	2,847	2
fabp1a Δ5'FR*	AATCCATAAATGGGGTCTGGATACAA<br>TTGTATCCAGACCCCATTTATGGATT	-	2
fabp1a ΔDR1*	AAACCTGGACGTGGATCAAAAGGAGT<br>ACTCCTTTTGATCCACGTCCAGGTTT	-	2
fabp1b.1 Δ5'FR*	TGGTTCGAGTTCGGGTTACGGATAAG<br>CTTATCCGTAACCCGAACTCGAACCA	-	2
fabp1b.1 ΔDR1*	AAACTACGGACGGAACCATGAATTGG<br>CCAATTCATGGTTCCGTCCGTAGTTT	-	2
fabp1 ΔPPRE-1*	ACATCTTCGACACGACCAGCTTTGATTCAAAACTTTTTTTCGGGGTTCCCTCACTTTTCAC<br>GTGAAAAGTGAGGGAACCCCGAAAAAAAGTTTTGAATCAAAGCTGGTCGTGTCGAAGATGT	-	2
fabp1 ΔPPRE-2*	TATAGTGTAGTACAGGAGTCTATAAACTGTAAAGTGGGAGTCTGTGTAGTGTAGTACAGGAGTC<br>GACTCCTGTACTACACTACACAGACTCCCACTTTACAGTTTATAGACTCCTGTACTACACTATA	-	2
fabp1a qRT-PCR	CCGGATGATGAGGTCGAGAAA<br>TCGCCCACAGTGAAGGAGTA	128	2
fabp1b.1 qRT-PCR	ACACTGGTCAACACTCTGACG<br>AAGTCTTGCGTGTGTTTGCT	98	1
fabp1b.2 qRT-PCR	ATCCAGATGCTGAGCGGAGA<br>ATGTTGTCAGCGGTGCTCAG	146	2
GAPDH qRT-PCR	GGCCGTTACAAGGGAGAAGT<br>GTGGACTCGACCACGTACAG	137	2

*SDM, primers used in site-directed mutagenesis

†Bold font denotes *Mlu* I (Fwd) and *Hind* III (Rev) restriction sites, underlined nucleotides denote mutagenized residues.
